# Supplementary material for: Renewable energy as a solution to climate change: Insights from a comprehensive study across nations
Source: PLoS One. 2024 Jun 20;19(6):e0299807. doi: 10.1371/journal.pone.0299807 (PMC11189203; doi:10.1371/journal.pone.0299807)
Supplement: S2 Appendix — (DOCX) [file pone.0299807.s002.docx]

# S2 Appendix: Descriptive statistics for Renewable Energy Consumption (% of final energy consumption) and CO_2_ emissions (millions of tons)

|  | **Variables** | | | | | | | | | | | |
| --- | --- | --- | --- | --- | --- | --- | --- | --- | --- | --- | --- | --- |
| **Country** | | **Development Category** |  | **Renewable energy consumption**  **(% of final energy consumption)** | | | |  | **CO_2_ emissions (millions of tons)** | | | |
|  |  |  |  | **Mean** | **SD** | **Min** | **Max** |  | **Mean** | **SD** | **Min.** | **Max** |
| Albania | | EiT |  | 39.3928 | 6.0914 | 31.62 | 55.9534 |  | 4.0819 | 1.1850 | 1.5430 | 5.9997 |
| Algeria | | DingE |  | 0.3041 | 0.1721 | 0.0565 | 0.58 |  | 122.5358 | 33.2426 | 82.2210 | 179.5048 |
| Andorra | | DE |  | 17.2376 | 2.0105 | 14.1001 | 20.3872 |  | 0.4981 | 0.0384 | 0.425024 | 0.5752 |
| Angola | | LD |  | 59.7834 | 10.6989 | 43.9367 | 76.8198 |  | 19.3190 | 7.5754 | 7.2684 | 30.3297 |
| Argentina | | DingE |  | 9.7493 | 1.1084 | 7.65 | 11.62 |  | 166.1591 | 23.7602 | 124.3923 | 191.7438 |
| Aruba | | DingE |  | 3.2145 | 3.4966 | 0.1579 | 8.3381 |  | 1.6268 | 0.8789 | 0.7072 | 2.8213 |
| Australia | | DE |  | 8.3968 | 1.0273 | 6.68 | 10.1 |  | 380.8406 | 33.5397 | 305.003 | 416.3566 |
| Austria | | DE |  | 29.6428 | 4.7233 | 22.56 | 36.6765 |  | 69.2874 | 4.5725 | 62.0375 | 79.0781 |
| Azerbaijan | | EiT |  | 2.5501 | 0.7322 | 1.3668 | 4.45 |  | 33.0743 | 2.9529 | 28.5698 | 39.1086 |
| Bahamas | | DingE |  | 1.4866 | 0.5607 | 0.38 | 3.54 |  | 2.0878 | 0.2673 | 1.6085 | 2.7040 |
| Bangladesh | | LD |  | 44.2049 | 13.1283 | 22.7834 | 63.8028 |  | 50.9864 | 23.8176 | 21.0417 | 93.1759 |
| Barbados | | DingE |  | 8.5083 | 4.6355 | 1.2927 | 16.8069 |  | 1.2478 | 0.2074 | 0.8066 | 1.6123 |
| Belarus | | EiT |  | 6.3718 | 1.6176 | 2.5278 | 8.6017 |  | 59.8387 | 2.8378 | 53.8441 | 64.1256 |
| Belgium | | DE |  | 5.2267 | 3.7457 | 1.1484 | 11.1559 |  | 113.9093 | 13.3637 | 90.3680 | 130.2124 |
| Belize | | DingE |  | 35.0180 | 4.8867 | 29.1 | 50.07 |  | 0.4863 | 0.1093 | 0.3078 | 0.7071 |
| Benin | | LD |  | 58.1811 | 14.8412 | 35.2126 | 94.7720 |  | 3.8987 | 2.2023 | 0.9563 | 7.7559 |
| Bhutan | | LD |  | 89.2533 | 3.7379 | 82.2 | 94.3718 |  | 0.6747 | 0.4296 | 0.2363 | 1.5221 |
| Bolivia | | DingE |  | 18.9028 | 10.1788 | 2.3722 | 33.5360 |  | 15.3435 | 5.4217 | 8.1712 | 23.7990 |
| Botswana | | DingE |  | 31.6017 | 7.1540 | 20.3849 | 47.1740 |  | 4.7141 | 1.4204 | 2.7553 | 7.5043 |
| Brazil | | DingE |  | 45.0272 | 1.9145 | 41.33 | 48.92 |  | 410.0133 | 82.6686 | 268.678 | 557.901 |
| Bulgaria | | DE |  | 12.1258 | 5.5935 | 3.1067 | 21.1133 |  | 48.6956 | 5.2023 | 36.9671 | 58.5016 |
| Burundi | | LD |  | 92.5784 | 3.3158 | 84.8 | 96.04 |  | 0.3386 | 0.1765 | 0.1539 | 0.7179 |
| Cambodia | | LD |  | 70.5002 | 10.8658 | 53.28 | 82.7662 |  | 6.2039 | 5.6061 | 1.4656 | 19.0286 |
| Canada | | DE |  | 21.9766 | 0.3915 | 21.18 | 22.69 |  | 558.9724 | 24.5520 | 491.3868 | 593.5158 |
| Cape Verde | | DingE |  | 25.8494 | 5.3318 | 19.1320 | 39.0786 |  | 0.4519 | 0.1361 | 0.1979 | 0.6677 |
| Chad | | LD |  | 83.7277 | 8.0599 | 71.3828 | 98.1385 |  | 1.1165 | 0.5936 | 0.4214 | 2.0154 |
| China | | DingE |  | 18.2726 | 8.1372 | 6.0396 | 30.5372 |  | 7207.025 | 2926.652 | 3357.909 | 11472.37 |
| Colombia | | DingE |  | 30.1239 | 2.0129 | 25.8003 | 33.7754 |  | 72.3061 | 15.2826 | 54.5693 | 98.7301 |
| Comoros | | LD |  | 63.3136 | 6.4726 | 49.5941 | 74.36 |  | 0.1606 | 0.0719 | 0.0770 | 0.3114 |
| Costa Rica | | DingE |  | 36.8333 | 3.6140 | 28.8905 | 42.4 |  | 6.8692 | 1.1530 | 4.6864 | 8.2295 |
| Croatia | | DE |  | 28.8561 | 3.1570 | 22.66 | 34.1265 |  | 19.9650 | 2.4150 | 16.8177 | 24.8629 |
| Curacao | | DingE |  | 0.9562 | 0.8801 | 0.07 | 3.02 |  | 4.5114 | 1.6262 | 0.2489 | 6.9250 |
| Cyprus | | DE |  | 6.5701 | 3.5643 | 3.03 | 12.1 |  | 7.3533 | 0.7082 | 5.8741 | 8.7173 |
| Czechia | | DE |  | 10.2703 | 4.0227 | 5.2415 | 16.6886 |  | 117.1297 | 11.8674 | 91.8539 | 135.0043 |
| Denmark | | DE |  | 20.9194 | 10.6402 | 7.2611 | 38.0982 |  | 48.6088 | 12.3157 | 28.2820 | 74.8686 |
| Dominican Republic | | DingE |  | 17.6562 | 2.0146 | 14 | 22.3363 |  | 21.0057 | 3.5330 | 15.1070 | 28.9221 |
| Ecuador | | DingE |  | 15.6919 | 2.6686 | 11.79 | 21.0557 |  | 31.7053 | 7.7238 | 18.2065 | 43.7314 |
| Egypt | | DingE |  | 6.4754 | 1.4095 | 4.2460 | 9.0030 |  | 182.528 | 52.8573 | 93.5687 | 260.1106 |
| El Salvador | | DingE |  | 32.6290 | 11.6070 | 15.2364 | 56.9374 |  | 6.1970 | 0.6301 | 4.4145 | 7.4229 |
| Eritrea | | LD |  | 22.4449 | 25.5085 | 3.54 | 74 |  | 0.6671 | 0.1256 | 0.4094 | 0.8985 |
| Estonia | | DE |  | 22.7052 | 4.7135 | 12.3234 | 31.3 |  | 16.7620 | 2.6184 | 9.34301 | 19.9664 |
| Eswatini | | DingE |  | 22.7051 | 4.7135 | 12.3240 | 31.3 |  | 1.0551 | 0.1278 | 0.7694 | 1.3996 |
| Ethiopia | | LD |  | 93.3751 | 2.3646 | 88.9 | 96.6201 |  | 8.1804 | 5.2138 | 2.5060 | 18.2978 |
| Fiji | | DingE |  | 38.2385 | 11.3224 | 20.8438 | 60.0612 |  | 1.0437 | 0.2328 | 0.7365 | 1.4704 |
| Finland | | DE |  | 35.2578 | 6.3686 | 26.8705 | 45.8 |  | 55.7898 | 9.7543 | 26.8705 | 72.6543 |
| France | | DE |  | 11.3819 | 2.2656 | 8.52 | 15.5 |  | 371.9753 | 40.0918 | 280.0315 | 416.1658 |
| French Polynesia | | DingE |  | 8.3426 | 1.3306 | 6.49 | 11.1942 |  | 0.7489 | 0.1661 | 0.4324 | 0.9498 |
| Gambia | | LD |  | 56.0115 | 4.7704 | 48 | 62.86 |  | 0.4035 | 0.1410 | 0.2162 | 0.6557 |
| Georgia | | EiT |  | 38.3352 | 10.3055 | 23.7724 | 56.76 |  | 6.6829 | 2.7208 | 2.2903 | 11.0098 |
| Germany | | DE |  | 9.7728 | 5.3911 | 2.2522 | 18.5213 |  | 837.935 | 80.5433 | 639.3811 | 958.7003 |
| Ghana | | DingE |  | 56.6995 | 13.5904 | 34.6715 | 79.7893 |  | 10.8537 | 4.9121 | 5.1536 | 21.3128 |
| Greece | | DE |  | 11.5403 | 4.3973 | 7.11 | 18.5 |  | 91.4362 | 17.6716 | 55.61028 | 114.5457 |
| Guatemala | | DingE |  | 64.8779 | 1.6369 | 61.35 | 67.45 |  | 12.4754 | 3.8390 | 6.5711 | 20.3271 |
| Guinea | | LD |  | 79.2877 | 6.3124 | 65.4 | 89.9303 |  | 2.3006 | 1.0232 | 1.1651 | 4.8418 |
| Guyana | | DingE |  | 29.2741 | 7.1452 | 11.4 | 38.6082 |  | 2.0018 | 0.4287 | 1.5096 | 3.1910 |
| Honduras | | DingE |  | 52.4361 | 5.3233 | 45.9 | 65.3815 |  | 7.7086 | 2.2963 | 3.8362 | 11.2321 |
| Hong Kong | | DingE |  | 0..2564 | 0.2577 | 0.0309 | 0.8586 |  | 39.9887 | 4.7916 | 28.9085 | 45.4918 |
| Hungary | | DE |  | 10.4398 | 4.9303 | 4.63 | 17.8164 |  | 54.4202 | 6.5354 | 43.6116 | 63.0248 |
| Iceland | | DE |  | 69.8621 | 10.2510 | 52.2532 | 86.2456 |  | 3.2239 | 0.3970 | 2.4596 | 3.8011 |
| India | | DingE |  | 40.5928 | 8.1944 | 27.3897 | 54.4841 |  | 1607.725 | 657.4508 | 761.4492 | 2709.684 |
| Indonesia | | DingE |  | 36.1144 | 9.8251 | 19.1 | 50.0981 |  | 416.0158 | 136.2238 | 212.4753 | 659.4357 |
| Iran | | DingE |  | 0.9178 | 0.2288 | 0.44 | 1.4 |  | 514.4456 | 148.1619 | 267.0457 | 748.8787 |
| Iraq | | DingE |  | 0.9351 | 0.6575 | 0.31 | 2.56 |  | 123.4545 | 49.8747 | 61.1694 | 213.4311 |
| Ireland | | DE |  | 5.4408 | 3.5345 | 1.9007 | 12.3 |  | 41.4238 | 4.2936 | 35.1532 | 48.1566 |
| Israel | | DingE |  | 5.7421 | 1.7572 | 2.88 | 9.08 |  | 60.3744 | 5.5233 | 49.8655 | 74.7848 |
| Italy | | DE |  | 10.8314 | 5.3674 | 4.4623 | 19.3504 |  | 424.3043 | 62.4584 | 302.2786 | 502.2554 |
| Japan | | DE |  | 4.9374 | 1.2431 | 3.5 | 7.69 |  | 1227.7 | 70.5257 | 1042.224 | 1315.569 |
| Jordan | | DingE |  | 3.2225 | 1.6696 | 1.69 | 8.17 |  | 20.3489 | 4.4613 | 13.2081 | 26.3280 |
| Kazakhstan | | EiT |  | 1.7675 | 0.4142 | 1.15 | 2.77 |  | 224.6158 | 61.7533 | 123.7217 | 331.8207 |
| Kenya | | DingE |  | 77.2556 | 4.0269 | 68.1 | 83.25 |  | 12.0296 | 4.0679 | 6.5871 | 19.8754 |
| Kyrgyzstan | | EiT |  | 26.9647 | 4.2571 | 21.8900 | 36 |  | 7.1648 | 2.1790 | 3.8614 | 11.1045 |
| Latvia | | DE |  | 36.7746 | 3.21756 | 32.0254 | 42.6 |  | 7.8109 | 0.6173 | 6.9941 | 9.2115 |
| Lebanon | | DingE |  | 5.3826 | 1.082 | 3.96 | 7.94 |  | 19.6690 | 4.7596 | 12.2547 | 27.8463 |
| Lesotho | | LD |  | 49.2409 | 6.5665 | 39.5 | 56.8799 |  | 2.1588 | 0.3522 | 1.7074 | 3.0924 |
| Liechtenstein | | DE |  | 53.0813 | 2.6427 | 49.1792 | 56.9833 |  | 0.1940 | 0.0316 | 0.1420 | 0.2311 |
| Lithuania | | DE |  | 21.9044 | 7.6725 | 10.3412 | 34.0502 |  | 13.9194 | 1.0544 | 11.8764 | 16.0306 |
| Luxembourg | | DE |  | 6.3413 | 4.6970 | 1.28 | 16.5 |  | 9.8831 | 1.2612 | 7.6760 | 12.0878 |
| Macao | | DE |  | 4.6783 | 4.36 | 0.18 | 16.61 |  | 1.5022 | 0.2453 | 1.1322 | 1.9987 |
| Malawi | | LD |  | 80.3431 | 2.8729 | 73 | 83.3 |  | 1.0802 | 1.0802 | 0.7907 | 1.5930 |
| Malaysia | | DingE |  | 4.1342 | 1.9211 | 1.96 | 9.0296 |  | 191.172 | 55.2284 | 108.2914 | 269.1557 |
| Maldives | | DingE |  | 1.7246 | 0.7136 | 0.6355 | 3.3185 |  | 0.9520 | 0.5617 | 0.2492 | 2.1182 |
| Mali | | LD |  | 80.8154 | 4.0889 | 74.8128 | 88.0725 |  | 2.1116 | 1.1349 | 0.5952 | 4.1698 |
| Malta | | DE |  | 2.4525 | 3.1841 | 0 | 9.22 |  | 2.3440 | 0.4795 | 1.3564 | 2.9605 |
| Mauritius | | LD |  | 16.6961 | 9.5035 | 2.5207 | 38.8345 |  | 3.4781 | 0.8678 | 1.8283 | 4.5360 |
| Mexico | | DingE |  | 10.3382 | 1.4453 | 8.3528 | 13.2618 |  | 441.1064 | 49.1461 | 331.5966 | 501.5688 |
| Micronesia | | DingE |  | 1.4322 | 0.2260 | 0.86 | 1.95 |  | 0.1487 | 0.0267 | 0.1026 | 0.2015 |
| Moldova | | EiT |  | 13.3401 | 9.9884 | 2.8426 | 28.0481 |  | 5.4637 | 1.8985 | 3.5730 | 11.6871 |
| Mongolia | | DingE |  | 4.1657 | 1.0897 | 2.97 | 6.51 |  | 20.4386 | 15.4818 | 7.4319 | 50.3159 |
| Morocco | | DingE |  | 14.3144 | 3.4066 | 9.9304 | 23 |  | 48.3180 | 13.0195 | 28.7503 | 70.5777 |
| Mozambique | | LD |  | 87.1809 | 6.2022 | 77.5587 | 94.1666 |  | 3.4833 | 2.5392 | 1.0918 | 8.1593 |
| Myanmar | | LD |  | 77.2129 | 8.7251 | 57.85 | 85.71 |  | 15.6750 | 9.4240 | 6.9073 | 36.3067 |
| Namibia | | DingE |  | 32.2805 | 2.1147 | 28.99 | 35.84 |  | 2.7331 | 0.9456 | 1.6048 | 4.2162 |
| Nepal | | LD |  | 86.2912 | 5.2069 | 74.27 | 91.7324 |  | 5.7960 | 4.2419 | 2.2233 | 14.8332 |
| Netherlands | | DE |  | 3.9591 | 2.2110 | 1.2770 | 8.54 |  | 168.1674 | 11.0886 | 137.8495 | 181.5267 |
| New Caledonia | | DingE |  | 5.8095 | 1.3481 | 3.7 | 9.0614 |  | 3.4105 | 1.3073 | 1.8100 | 5.4963 |
| New Zealand | | DE |  | 30.0270 | 1.3370 | 27.13 | 32.65 |  | 34.4029 | 2.5193 | 28.0035 | 37.5092 |
| Nicaragua | | DingE |  | 54.2709 | 4.6148 | 47.7449 | 64.5360 |  | 4.3559 | 0.7495 | 2.7551 | 5.4613 |
| Niger | | LD |  | 83.7660 | 5.0753 | 72.71 | 90.9116 |  | 1.3123 | 0.7470 | 0.5619 | 2.6989 |
| Nigeria | | DingE |  | 84.5118 | 2.3177 | 80.64 | 88.68 |  | 94.2053 | 31.2604 | 33.4167 | 136.9868 |
| North Macedonia | | EiT |  | 18.4860 | 2.5952 | 14.2069 | 23.91 |  | 9.4535 | 1.9124 | 6.6495 | 12.5711 |
| Norway | | DE |  | 58.5484 | 1.5665 | 56.23 | 62.37 |  | 43.4122 | 1.7054 | 38.508 | 45.691 |
| Pakistan | | DingE |  | 47.5712 | 3.3161 | 42.09 | 53.1229 |  | 147.537 | 42.0642 | 83.6144 | 229.5124 |
| Panama | | DingE |  | 24.8171 | 5.9277 | 16.5191 | 37.3484 |  | 8.2846 | 2.7414 | 3.6530 | 13.4195 |
| Paraguay | | DingE |  | 65.0470 | 3.8034 | 59.2011 | 70.7591 |  | 5.2219 | 1.6912 | 3.6061 | 8.5766 |
| Peru | | DingE |  | 32.6676 | 4.6972 | 27.12 | 41.23 |  | 40.1312 | 11.9536 | 24.2244 | 57.6799 |
| Philippines | | DingE |  | 31.9915 | 2.8521 | 26.73 | 38.942 |  | 90.2898 | 28.4385 | 59.9504 | 145.2317 |
| Poland | | DE |  | 8.9842 | 2.3283 | 5.8650 | 12.6391 |  | 329.4865 | 17.5325 | 303.5231 | 377.5779 |
| Portugal | | DE |  | 24.5422 | 3.6955 | 18.07 | 30.46 |  | 56.4642 | 8.3176 | 40.7972 | 69.7180 |
| Romania | | DE |  | 19.5787 | 4.6105 | 8.6027 | 26.5766 |  | 93.8371 | 15.2085 | 74.1380 | 128.9356 |
| Russia | | EiT |  | 3.4468 | 0.2162 | 3.1568 | 3.8707 |  | 1598.064 | 77.6851 | 1464.543 | 1755.547 |
| Saudi Arabia | | DingE |  | 0.0123 | 0.0053 | 0.0090 | 0.03 |  | 453.4265 | 161.1419 | 207.2401 | 678.8493 |
| Senegal | | LD |  | 43.7310 | 5.3046 | 36.15 | 53.1779 |  | 6.8324 | 3.2791 | 3.1824 | 13.5979 |
| Seychelles | | DingE |  | 1.2125 | 0.4771 | 0.71 | 2.9636 |  | 0.4117 | 0.1104 | 0.1979 | 0.6009 |
| Singapore | | DingE |  | 0.5540 | 0.1137 | 0.33 | 0.84 |  | 45.3835 | 8.2798 | 29.9095 | 59.9907 |
| Slovenia | | DE |  | 18.1376 | 3.9874 | 10.2327 | 23.9304 |  | 15.5613 | 1.4078 | 12.5493 | 18.2784 |
| South Africa | | DingE |  | 12.4075 | 3.1365 | 7.7505 | 18.2213 |  | 428.0771 | 42.171 | 356.3048 | 494.9997 |
| Spain | | DE |  | 12.2895 | 4.1483 | 7.3 | 18.3805 |  | 292.1407 | 42.4415 | 213.3397 | 370.0658 |
| Sri Lanka | | DingE |  | 59.3949 | 5.5667 | 49.33 | 69.9385 |  | 14.0580 | 5.3118 | 5.8143 | 23.0305 |
| Sudan | | LD |  | 69.2061 | 8.9756 | 55.2399 | 83.6105 |  | 12.8632 | 6.4617 | 3.9029 | 22.4316 |
| Suriname | | DingE |  | 21.0595 | 6.1568 | 11.29 | 30.0900 |  | 2.1574 | 0.3971 | 1.5378 | 2.8855 |
| Sweden | | DE |  | 43.2014 | 7.2776 | 31.3561 | 54.7580 |  | 50.4212 | 7.4820 | 35.8490 | 63.5218 |
| Switzerland | | DE |  | 20.5290 | 2.7013 | 17.1169 | 24.99 |  | 42.0016 | 3.4319 | 34.2410 | 45.7786 |
| Syria | | DingE |  | 1.6344 | 0.5798 | 0.58 | 3.07 |  | 44.4684 | 12.6427 | 26.1598 | 66.8767 |
| Tajikistan | | EiT |  | 54.8901 | 8.6577 | 38.56 | 64.58 |  | 4.1019 | 2.7465 | 1.8772 | 10.3364 |
| Tanzania | | LD |  | 89.3004 | 3.5692 | 83.7484 | 94.2663 |  | 6.8524 | 3.7558 | 2.4339 | 13.0589 |
| Thailand | | DingE |  | 22.0974 | 1.3770 | 19.89 | 24.41 |  | 236.1542 | 47.2151 | 156.0273 | 293.0742 |
| Togo | | LD |  | 76.1843 | 4.5659 | 63.38 | 82.05 |  | 1.8021 | 0.4960 | 0.8790 | 2.7408 |
| Tunisia | | DingE |  | 13.5889 | 1.0858 | 11.82 | 16.07 |  | 24.1165 | 4.9001 | 15.5469 | 31.5828 |
| Turkey | | DingE |  | 15.1492 | 3.6447 | 9.7089 | 22.0984 |  | 306.4792 | 83.4289 | 181.4818 | 446.2042 |
| Uganda | | LD |  | 93.2147 | 1.8819 | 90.07 | 95.9711 |  | 3.0441 | 1.7070 | 0.9255 | 6.01181 |
| Ukraine | | EiT |  | 2.9851 | 2.0756 | 0.8979 | 7.44 |  | 288.8599 | 48.4240 | 201.8577 | 389.8646 |
| United Kingdom | | DE |  | 4.3350 | 3.8526 | 0.85 | 12.24 |  | 498.0455 | 84.3705 | 326.2632 | 586.7607 |
| United States of America | | DE |  | 7.2473 | 2.1698 | 4.5143 | 10.7077 |  | 5622.654 | 368.9432 | 4715.691 | 6137.604 |
| Uzbekistan | | EiT |  | 1.2816 | 0.3058 | 0.72 | 1.75 |  | 115.4256 | 7.5832 | 102.6637 | 127.5183 |
| Venezuela | | DingE |  | 14.4931 | 1.1360 | 12.01 | 16.64 |  | 152.456 | 33.4984 | 76.4675 | 198.427 |
| Vietnam | | DingE |  | 41.2968 | 14.7224 | 17.5273 | 65.1258 |  | 140.4681 | 95.1542 | 28.6967 | 341.0049 |
| Zambia | | LD |  | 86.8868 | 2.8641 | 81.89 | 90.32 |  | 3.7146 | 2.0984 | 1.7776 | 7.7472 |
| Zimbabwe | | DingE |  | 77.2262 | 6.2561 | 64.4125 | 86.0492 |  | 11.4824 | 2.0679 | 7.7201 | 15.7278 |

Note: SD, Min., and Max. represent Observations, Standard Deviation, Minimum value, and Maximum value, respectively. All countries have 27 observations each.

DE, DingE, EiT, LD represent Developed Economies, Developing Economies, Economies in Transition, Least Developed Economies respectively.
