# Supplementary material for: Renewable energy as a solution to climate change: Insights from a comprehensive study across nations
Source: PLoS One. 2024 Jun 20;19(6):e0299807. doi: 10.1371/journal.pone.0299807 (PMC11189203; doi:10.1371/journal.pone.0299807)
Supplement: S3 Appendix — (DOCX) [file pone.0299807.s003.docx]

# S3 Appendix: Difference of averages of CO_2_ Emissions from 1995-2004 and 2012-2021

| **Country** | **Average of CO_2_ from 1995-2004** | **Average of CO_2_ from 2012-2021** | **Percentage Change** | |
| --- | --- | --- | --- | --- |
| **Developed Countries** | | | |  |
| Andorra | 0.501 | 0.471 | -6% | 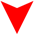 |
| Australia | 343.515 | 404.915 | 18% | 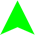 |
| Austria | 69.459 | 66.351 | -4% | 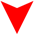 |
| Belgium | 127.096 | 98.71 | -22% | 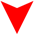 |
| Bulgaria | 51.257 | 44.301 | -14% | 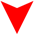 |
| Canada | 544.487 | 566.661 | 4% | 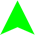 |
| Croatia | 20.02 | 18.002 | -10% | 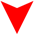 |
| Cyprus | 6.858 | 7.222 | 5% | 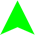 |
| Czechia | 127.333 | 103.797 | -18% | 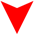 |
| Denmark | 60.335 | 34.98 | -42% | 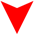 |
| Estonia | 17.009 | 15.863 | -7% | 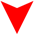 |
| Finland | 62.922 | 44.969 | -29% | 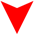 |
| France | 405.993 | 327.103 | -19% | 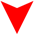 |
| Germany | 914.855 | 759.437 | -17% | 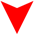 |
| Greece | 99.831 | 72.234 | -28% | 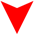 |
| Hungary | 60.808 | 47.18 | -22% | 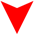 |
| Iceland | 2.788 | 3.494 | 25% | 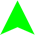 |
| Ireland | 42.606 | 37.956 | -11% | 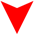 |
| Italy | 468.856 | 351.406 | -25% | 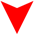 |
| Japan | 1254.93 | 1186.107 | -5% | 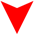 |
| Latvia | 8.061 | 7.351 | -9% | 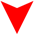 |
| Liechtenstein | 0.22 | 0.159 | -28% | 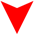 |
| Lithuania | 13.876 | 13.62 | -2% | 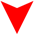 |
| Luxembourg | 9.29 | 9.45 | 2% | 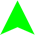 |
| Macao | 1.529 | 1.475 | -4% | 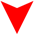 |
| Malta | 2.628 | 1.855 | -29% | 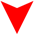 |
| Netherlands | 175.639 | 156.907 | -11% | 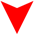 |
| New Zealand | 32.316 | 35.335 | 9% | 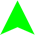 |
| Norway | 42.288 | 43.783 | 4% | 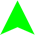 |
| Poland | 335.915 | 322.291 | -4% | 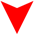 |
| Portugal | 61.968 | 48.567 | -22% | 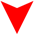 |
| Romania | 105.937 | 79.369 | -25% | 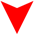 |
| Slovenia | 16.019 | 14.119 | -12% | 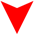 |
| Spain | 301.802 | 256.525 | -15% | 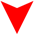 |
| Sweden | 57.803 | 42.105 | -27% | 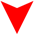 |
| Switzerland | 44.17 | 38.354 | -13% | 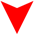 |
| United Kingdom | 569.814 | 403.067 | -29% | 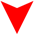 |
| United States | 5829.835 | 5255.338 | -10% | 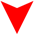 |
| **Developing Countries** | | | |  |
| Algeria | 91.13715 | 161.517 | 77% | 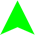 |
| Argentina | 137.9334 | 185.261 | 34% | 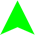 |
| Aruba | 1.620587 | 0.917 | -43% | 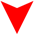 |
| Bahamas | 1.941187 | 2.346 | 21% | 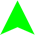 |
| Barbados | 1.083147 | 1.266 | 17% | 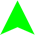 |
| Belize | 0.389117 | 0.585 | 50% | 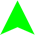 |
| Bolivia | 10.21702 | 21.627 | 112% | 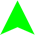 |
| Botswana | 3.441962 | 6.348 | 84% | 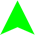 |
| Brazil | 324.9746 | 499.153 | 54% | 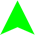 |
| Cape Verde | 0.301914 | 0.558 | 85% | 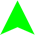 |
| China | 3881.646 | 10290.07 | 165% | 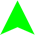 |
| Coasta Rica | 5.580526 | 7.743 | 39% | 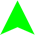 |
| Colombia | 58.2253 | 89.621 | 54% | 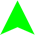 |
| Curacao | 4.034017 | 4.451 | 10% | 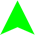 |
| Dominican Republic | 18.223 | 24.589 | 35% | 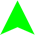 |
| Ecuador | 23.23835 | 39.662 | 71% | 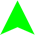 |
| Egypt | 122.7966 | 236.152 | 92% | 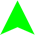 |
| El Salvador | 5.643454 | 6.548 | 16% | 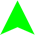 |
| Eswatini | 1.078315 | 1.056 | -2% |  |
| Fiji | 0.873005 | 1.254 | 43% | 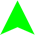 |
| French Polynesia | 0.567554 | 0.882 | 55% | 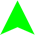 |
| Ghana | 6.410197 | 16.6 | 159% | 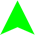 |
| Guatemala | 9.09447 | 16.48 | 81% | 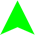 |
| Guyana | 1.76788 | 2.4 | 36% | 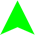 |
| Honduras | 5.175985 | 10.019 | 94% | 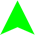 |
| Hong Kong | 37.44879 | 40.84 | 9% | 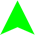 |
| India | 944.8742 | 2365.841 | 150% | 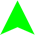 |
| Indonesia | 277.977 | 564.412 | 103% | 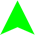 |
| Iran | 351.7236 | 665.255 | 89% | 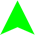 |
| Iraq | 80.22431 | 181.902 | 127% | 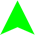 |
| Israel | 57.0371 | 60.955 | 7% | 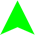 |
| Jordan | 15.25103 | 25.059 | 64% | 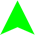 |
| Kenya | 8.60778 | 16.498 | 92% | 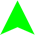 |
| Lebanon | 15.66764 | 25.056 | 60% | 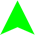 |
| Malaysia | 129.4922 | 248.853 | 92% | 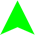 |
| Maldives | 0.426856 | 1.565 | 267% | 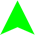 |
| Mexico | 392.0227 | 465.248 | 19% | 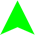 |
| Micronesia | 0.174773 | 0.142 | -18% | 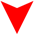 |
| Mongolia | 7.836114 | 38.366 | 390% | 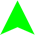 |
| Morocco | 33.72259 | 61.608 | 83% | 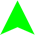 |
| Namibia | 1.785101 | 3.795 | 113% | 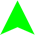 |
| New Caledonia | 2.165984 | 4.913 | 127% | 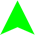 |
| Nicaragua | 3.605262 | 5.025 | 39% | 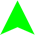 |
| Nigeria | 64.70858 | 118.709 | 83% | 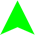 |
| Pakistan | 103.9636 | 189.231 | 82% | 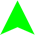 |
| Panama | 5.455632 | 11.247 | 106% | 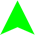 |
| Paraguay | 3.979545 | 7.043 | 77% | 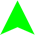 |
| Peru | 27.49737 | 51.03 | 86% | 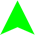 |
| Philippines | 68.34905 | 122.328 | 79% | 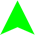 |
| Saudi Arabia | 280.9481 | 630.582 | 124% | 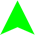 |
| Seychelles | 0.301914 | 0.523 | 73% | 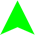 |
| Singapore | 48.28931 | 41.91 | -13% | 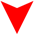 |
| South Africa | 382.0779 | 450.594 | 18% | 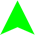 |
| Sri Lanka | 9.106658 | 19.974 | 119% | 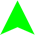 |
| Suriname | 1.968359 | 2.473 | 26% | 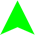 |
| Syria | 47.14559 | 31.38 | -33% | 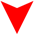 |
| Thailand | 182.7738 | 285.244 | 56% | 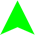 |
| Tunisia | 18.84305 | 29.289 | 55% | 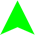 |
| Turkey | 215.8892 | 397.067 | 84% | 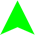 |
| Venezuela | 158.7381 | 135.673 | -15% | 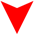 |
| Vietnam | 54.56469 | 242.671 | 345% | 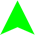 |
| Zimbabwe | 13.19692 | 11.207 | -15% | 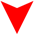 |
| **Economies in Transition** | | | |  |
| Albania | 2.886 | 5.032 | 74% | 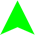 |
| Azerbaijan | 30.523 | 35.302 | 16% |  |
| Belarus | 57.836 | 60.884 | 5% |  |
| Georgia | 3.962 | 9.788 | 147% |  |
| Kazakhstan | 158.141 | 290.438 | 84% |  |
| Kyrgyzstan | 5.087 | 9.697 | 91% |  |
| Moldova | 6.194 | 5.112 | -17% |  |
| North Macedonia | 11.428 | 7.399 | -35% |  |
| Russia | 1521.989 | 1664.016 | 9% |  |
| Tajikistan | 2.347 | 6.875 | 193% |  |
| Ukraine | 321.009 | 240.219 | -25% |  |
| Uzbekistan | 117.724 | 113.643 | -3% |  |
| **Least Developed Countries** | | | |  |
| Angola | 10.46 | 23.8687 | 128% |  |
| Bangladesh | 27.287 | 77.6750 | 185% |  |
| Benin | 1.618 | 6.1990 | 283% |  |
| Bhutan | 0.344 | 1.1749 | 242% |  |
| Burundi | 0.228 | 0.5296 | 132% |  |
| Cambodia | 1.951 | 11.9984 | 515% |  |
| Chad | 0.524 | 1.7965 | 243% |  |
| Comoros | 0.104 | 0.2347 | 129% |  |
| Eritrea | 0.705 | 0.7127 | 1% |  |
| Ethiopia | 3.67 | 14.1438 | 285% |  |
| Gambia | 0.266 | 0.5608 | 111% |  |
| Guinea | 1.461 | 3.2624 | 123% |  |
| Lesotho | 1.839 | 2.4098 | 31% |  |
| Malawi | 0.89 | 1.3129 | 48% |  |
| Mali | 0.998 | 3.4063 | 242% |  |
| Mauritius | 2.496 | 4.3045 | 73% |  |
| Mozambique | 1.378 | 6.4031 | 365% |  |
| Myanmar | 8.927 | 24.8839 | 179% |  |
| Nepal | 2.698 | 10.3971 | 285% |  |
| Niger | 0.683 | 2.2227 | 225% |  |
| Senegal | 3.997 | 10.4994 | 163% |  |
| Sudan | 5.886 | 19.8306 | 237% |  |
| Tanzania | 2.974 | 11.1960 | 276% |  |
| Togo | 1.381 | 2.0734 | 50% |  |
| Uganda | 1.282 | 4.9092 | 283% |  |
| Zambia | 2.019 | 6.1340 | 204% |  |
