# Supplementary material for: Renewable energy as a solution to climate change: Insights from a comprehensive study across nations
Source: PLoS One. 2024 Jun 20;19(6):e0299807. doi: 10.1371/journal.pone.0299807 (PMC11189203; doi:10.1371/journal.pone.0299807)
Supplement: S4 Appendix — (DOCX) [file pone.0299807.s004.docx]

# S4 Appendix: Difference of averages of Renewable Energy Consumption from 1995-2004 and 2012-2021

| **Country** | **Average of REC from 1995-2004** | **Average of REC from**  **2012-2021** | **Percentage Change** | |
| --- | --- | --- | --- | --- |
| **Developed Countries** | | | |  |
| Andorra | 15.06 | 19.27 | 28% | 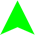 |
| Australia | 8.264 | 9.316 | 13% | 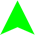 |
| Austria | 24.777 | 34.878 | 41% | 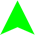 |
| Belgium | 1.489 | 9.599 | 545% | 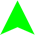 |
| Bulgaria | 6.561 | 18.41 | 181% | 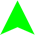 |
| Canada | 21.614 | 22.335 | 3% | 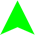 |
| Croatia | 27.931 | 31.659 | 13% | 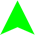 |
| Cyprus | 3.229 | 10.794 | 234% | 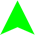 |
| Czechia | 6.2 | 14.922 | 141% | 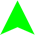 |
| Denmark | 10.133 | 33.153 | 227% | 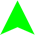 |
| Estonia | 18.738 | 27.631 | 47% | 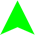 |
| Finland | 29.361 | 42.816 | 46% | 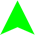 |
| France | 9.604 | 14.006 | 46% | 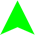 |
| Germany | 3.749 | 15.491 | 313% | 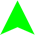 |
| Greece | 7.727 | 16.948 | 119% | 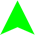 |
| Hungary | 5.124 | 15.704 | 206% | 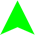 |
| Iceland | 58.812 | 80.231 | 36% | 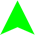 |
| Ireland | 2.067 | 9.511 | 360% | 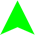 |
| Italy | 5.21 | 16.936 | 225% | 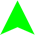 |
| Japan | 3.907 | 6.307 | 61% | 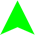 |
| Latvia | 34.503 | 40.379 | 17% | 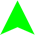 |
| Liechtenstein | 50.625 | 55.719 | 10% | 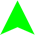 |
| Lithuania | 15.014 | 30.662 | 104% | 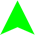 |
| Luxembourg | 3.822 | 11.028 | 189% | 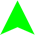 |
| Macao | 0.801 | 9.089 | 1035% | 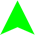 |
| Malta | 0.031 | 6.173 | 19813% | 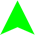 |
| Netherlands | 1.684 | 6.366 | 278% | 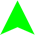 |
| New Zealand | 29.028 | 30.876 | 6% | 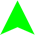 |
| Norway | 58.799 | 59.164 | 1% | 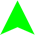 |
| Poland | 6.744 | 11.668 | 73% | 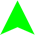 |
| Portugal | 21.628 | 27.967 | 29% | 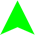 |
| Romania | 14.739 | 23.952 | 63% | 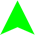 |
| Slovenia | 14.246 | 21.654 | 52% | 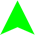 |
| Spain | 8.529 | 17.046 | 100% | 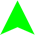 |
| Sweden | 35.444 | 51.183 | 44% | 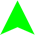 |
| Switzerland | 18.055 | 23.698 | 31% | 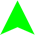 |
| United Kingdom | 0.986 | 8.816 | 794% | 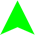 |
| United States | 5 | 9.713 | 94% | 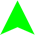 |
| **Developing Countries** | | | |  |
| Algeria | 0.461 | 0.115 | -75% | 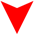 |
| Argentina | 10.714 | 9.569 | -11% | 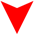 |
| Aruba | 0.173 | 7.288 | 4113% | 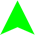 |
| Bahamas | 1.324 | 1.277 | -4% | 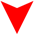 |
| Barbados | 13.225 | 3.618 | -73% | 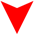 |
| Belize | 34.216 | 36.267 | 6% | 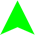 |
| Bolivia | 30.185 | 8.07 | -73% | 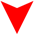 |
| Botswana | 39.574 | 25.01 | -37% | 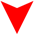 |
| Brazil | 43.948 | 44.823 | 2% | 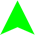 |
| Cape Verde | 30.849 | 23.404 | -24% | 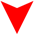 |
| China | 27.946 | 11.402 | -59% | 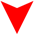 |
| Coasta Rica | 33.402 | 37.532 | 12% | 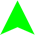 |
| Colombia | 29.231 | 31.232 | 7% | 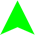 |
| Curacao | 0.287 | 1.938 | 575% | 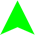 |
| Dominican Republic | 19.574 | 15.699 | -20% | 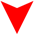 |
| Ecuador | 18.229 | 14.203 | -22% | 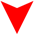 |
| Egypt | 8.128 | 5.164 | -36% | 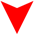 |
| El Salvador | 43.517 | 22.29 | -49% | 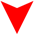 |
| Eswatini | 18.738 | 27.631 | 47% | 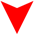 |
| Fiji | 49.66 | 28.207 | -43% | 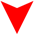 |
| French Polynesia | 8.552 | 7.5 | -12% | 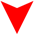 |
| Ghana | 71.679 | 42.834 | -40% | 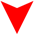 |
| Guatemala | 64.13 | 65.062 | 1% | 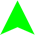 |
| Guyana | 34.736 | 21.401 | -38% | 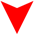 |
| Honduras | 55.765 | 49.741 | -11% | 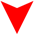 |
| Hong Kong | 0.405 | 0.176 | -57% | 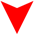 |
| India | 49.546 | 32.375 | -35% | 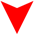 |
| Indonesia | 45.839 | 25.002 | -45% | 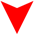 |
| Iran | 0.758 | 0.981 | 29% | 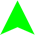 |
| Iraq | 0.358 | 0.95 | 165% | 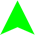 |
| Israel | 6.054 | 3.89 | -36% | 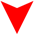 |
| Jordan | 2.104 | 4.82 | 129% | 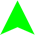 |
| Kenya | 80.211 | 72.955 | -9% | 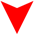 |
| Lebanon | 5.352 | 4.827 | -10% | 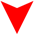 |
| Malaysia | 5.654 | 3.666 | -35% | 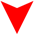 |
| Maldives | 2.47 | 1.109 | -55% | 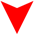 |
| Mexico | 11.858 | 9.359 | -21% | 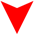 |
| Micronesia | 1.334 | 1.531 | 15% | 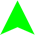 |
| Mongolia | 4.735 | 3.301 | -30% | 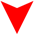 |
| Morocco | 16.885 | 10.821 | -36% | 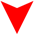 |
| Namibia | 33.617 | 30.168 | -10% | 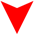 |
| New Caledonia | 6.542 | 5.049 | -23% | 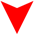 |
| Nicaragua | 58.342 | 50.716 | -13% | 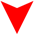 |
| Nigeria | 85.738 | 82.119 | -4% | 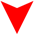 |
| Pakistan | 51.106 | 44.628 | -13% | 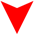 |
| Panama | 31.194 | 20.637 | -34% | 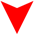 |
| Paraguay | 68.55 | 60.982 | -11% | 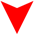 |
| Peru | 35.885 | 28.063 | -22% | 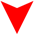 |
| Philippines | 33.471 | 29.791 | -11% | 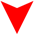 |
| Saudi Arabia | 0.012 | 0.015 | 25% | 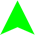 |
| Seychelles | 1.448 | 1.205 | -17% | 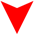 |
| Singapore | 0.482 | 0.662 | 37% | 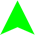 |
| South Africa | 15.924 | 9.803 | -38% | 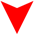 |
| Sri Lanka | 63.976 | 53.525 | -16% | 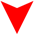 |
| Suriname | 26.634 | 14.079 | -47% | 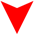 |
| Syria | 1.868 | 1.485 | -21% | 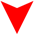 |
| Thailand | 20.979 | 23.333 | 11% | 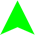 |
| Tunisia | 14.324 | 12.433 | -13% | 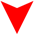 |
| Turkey | 19.234 | 12.21 | -37% | 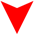 |
| Venezuela | 13.815 | 14.558 | 5% | 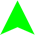 |
| Vietnam | 57.053 | 26.707 | -53% | 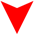 |
| Zimbabwe | 70.794 | 81.497 | 15% | 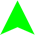 |
| **Economies in Transition** | | | |  |
| Albania | 43.663 | 38.087 | -13% |  |
| Azerbaijan | 2.19 | 2.26 | 3% |  |
| Belarus | 4.759 | 7.385 | 55% |  |
| Georgia | 49.771 | 27.87 | -44% |  |
| Kazakhstan | 2.006 | 1.64 | -18% |  |
| Kyrgyzstan | 31.052 | 23.893 | -23% |  |
| Moldova | 4.722 | 24.913 | 428% |  |
| North Macedonia | 16.533 | 20.43 | 24% |  |
| Russia | 3.613 | 3.27 | -9% |  |
| Tajikistan | 61.223 | 45.371 | -26% |  |
| Ukraine | 1.165 | 5.225 | 348% |  |
| Uzbekistan | 1.192 | 1.496 | 26% |  |
| **Least Developed Countries** | | | |  |
| Angola | 71.4310 | 49.9982 | -30% |  |
| Bangladesh | 58.0548 | 29.9455 | -48% |  |
| Benin | 73.9628 | 45.6782 | -38% |  |
| Bhutan | 92.1789 | 84.8390 | -8% |  |
| Burundi | 94.9075 | 88.9383 | -6% |  |
| Cambodia | 81.4842 | 58.5645 | -28% |  |
| Chad | 92.2792 | 76.5705 | -17% |  |
| Comoros | 61.7282 | 61.2564 | -1% |  |
| Eritrea | 36.9926 | 19.1706 | -48% |  |
| Ethiopia | 95.4523 | 90.7236 | -5% |  |
| Gambia | 59.6694 | 51.3397 | -14% |  |
| Guinea | 85.6041 | 73.1887 | -15% |  |
| Lesotho | 54.0377 | 41.8866 | -22% |  |
| Malawi | 82.1115 | 77.8190 | -5% |  |
| Mali | 85.1038 | 77.6367 | -9% |  |
| Mauritius | 26.1822 | 8.8932 | -66% |  |
| Mozambique | 93.2326 | 80.2208 | -14% |  |
| Myanmar | 82.6327 | 67.6240 | -18% |  |
| Nepal | 89.8240 | 80.5903 | -10% |  |
| Niger | 88.6081 | 78.7250 | -11% |  |
| Senegal | 47.0836 | 39.7582 | -16% |  |
| Sudan | 79.7331 | 61.2434 | -23% |  |
| Tanzania | 92.9106 | 85.0534 | -8% |  |
| Togo | 77.37923 | 77.1613 | -0.28% |  |
| Uganda | 95.040845 | 91.1147 | -4% |  |
| Zambia | 88.91305 | 83.4472 | -6% |  |
