# Supplementary material for: Renewable energy as a solution to climate change: Insights from a comprehensive study across nations
Source: PLoS One. 2024 Jun 20;19(6):e0299807. doi: 10.1371/journal.pone.0299807 (PMC11189203; doi:10.1371/journal.pone.0299807)
Supplement: S5 Appendix — (DOCX) [file pone.0299807.s005.docx]

# S5 Appendix: Results for fixed and random effect models for each economic development category

|  | **All Countries** | | **Developed** | | **Developing** | | **Economies in Transition** | | **Least Developed** | |
| --- | --- | --- | --- | --- | --- | --- | --- | --- | --- | --- |
|  | **FE** | **REf** | **FE** | **REf** | **FE** | **REf** | **FE** | **REf** | **FE** | **REf** |
| Intercept | 434.8364*** | 426.7438*** | 370.7557*** | 371.1469** | 701.6391*** | 656.2831*** | 205.0485*** | 205.3579 | 31.7621*** | 30.9045*** |
| REC | -7.4484 | -7.1855 | -3.1729** | -3.1933*** | -19.9367 | -18.0321 | -0.4945 | -0.5122* | -0.3609* | -0.3487* |
|  | (5.0198) | (4.6666) | (1.1605) | (1.1699) | (14.4701) | (13.0793) | (0.3035) | (0.3081) | (0.1851) | (0.1806) |
| N | 3726 | | 1026 | | 1674 | | 324 | | 702 | |
| No. of Countries | 138 | | 38 | | 62 | | 12 | | 26 | |
| R^2^ Within | 0.0280 | | 0.0488 | | 0.0690 | | 0.0070 | | 0.2894 | |
| R^2^ Between | 0.0218 | | 0.0449 | | 0.0011 | | 0.1297 | | 0.0518 | |
| R^2^ Overall | 0.0219 | | 0.0431 | | 0.0027 | | 0.1194 | | 0.0803 | |

Note: ***, **, * represent 1%, 5% and 10% significance level, respectively. All categories cover a period of 27 years.

Source: Authors’ calculations based on data from World Bank.
