# Supplementary material for: Renewable energy as a solution to climate change: Insights from a comprehensive study across nations
Source: PLoS One. 2024 Jun 20;19(6):e0299807. doi: 10.1371/journal.pone.0299807 (PMC11189203; doi:10.1371/journal.pone.0299807)
Supplement: S6 Appendix — (DOCX) [file pone.0299807.s006.docx]

# S6 Appendix: Regression results for individual countries

| **Country** |  | **Simple Regression** | **Multiple Regression**  **(Order 2)** | | **Multiple Regression (Quadratic)** | | **Multiple Regression (Polynomial)** | |
| --- | --- | --- | --- | --- | --- | --- | --- | --- |
| **Developed economies** | | | | | | | | |
| Andorra | Intercept  RE  RE^2^  RE^3^  RE^4^  R^2^ / R^2^ Adjusted | 0.5818***  -0.0049  0.0648 / 0.0274 | | -2.0322***  0.3060***  -0.0091***  0.6649 / 0.6370 | | -13.0959***  2.2701***  -0.1244***  0.0022***  0.7616 / 0.7305 | | 12.1805  -3.6475  0.3922  -0.01770  0.0003  0.7650 / 0.7223 |
| Australia | Intercept  RE  RE^2^  RE^3^  RE^4^  R^2^ / R^2^ Adjusted | 368.2556***  1.4988  0.0020/-0.0379 | | 1611.0030***  -306.7489***  18.8129***  0.2856 / 0.2260 | | -783.0340  -818.8508  80.7130  -2.4698  0.2986/ 0.2071 | | -54658.5  26599.85  -4771.706  376.5399  -11.0286  0.3561 / 0.2390 |
| Austria | Intercept  RE  RE^2^  RE^3^  RE^4^  R^2^ / R^2^ Adjusted | 84.5110***  -0.5134***  0.2814/0.2526 | | 63.5445  0.9347  -0.0244  0.2873 / 0.2279 | | 813.4630**  -75.9166  2.5950  -0.0294  0.4373/ 0.3639 | | 4649.989**  -610.1231*  30.1739*  -0.6563*  0.00529*  0.5045 / 0.4144 |
| Belgium | Intercept  RE  RE^2^  RE^3^  RE^4^  R^2^ / R^2^ Adjusted | 132.0671***  -3.4734***  0.9482/0.9461 | | 133.7307***  -4.4530***  0.0846  0.9507 / 0.9466 | | 130.0610***  -1.2724  -0.5521  0.0352  0.9539/ 0.9479 | | 122.7198***  7.1259  -3.3743  0.3862  -0.0144  0.9673/ 0.9495 |
| Bulgaria | Intercept  RE  RE^2^  RE^3^  RE^4^  R^2^ / R^2^ Adjusted | 57.4737***  -0.7239***  0.6058 / 0.5901 | | 57.3214***  -0.6930  -0.0013  0.6059 / 0.5730 | | 69.7288***  -5.0964***  0.4319***  -0.0123***  0.7017/ 0.6628 | | 90.8627***  -15.4553**  2.0198**  -0.1074**  0.0019*  0.7426 / 0.6958 |
| Canada | Intercept  RE  RE^2^  RE^3^  RE^4^  R^2^ / R^2^ Adjusted | 320.8862  10.8336  0.0298 / -0.0090 | | -3754.584  381.9718  -8.4470  0.0333 / -0.0473 | | 598592  -82081.98  3753.884  -57.2038  0.0546/ -0.0687 | | 142888.8  0  -1788.449  109.0616  -1.8698  0.0546 / -0.0688 |
| Croatia | Intercept  RE  RE^2^  RE^3^  RE^4^  R^2^ / R^2^ Adjusted | 39.4947***  -0.6768***  0.7828 / 0.7741 | | 77.3316***  -3.3391**  0.0463**  0.8187 / 0.8036 | | -145.898  20.5597  -0.7996  0.0099  0.8353 / 0.8139 | | -455.458  64.9597  -3.1724  0.0659  -0.0005  0.8357 / 0.8058 |
| Cyprus | Intercept  RE  RE^2^  RE^3^  RE^4^  R^2^ / R^2^ Adjusted | 7.2715***  0.0125  0.0039 / -0.0359 | | 5.8177***  0.5390*  -0.0362*  0.1341 / 0.0619 | | -2.1890  4.6599***  -0.6388***  0.0264***  0.4728/ 0.4040 | | -9.4282  9.4083*  -1.7045  0.1247  -0.0032  0.4900 / 0.3973 |
| Czechia | Intercept  RE  RE^2^  RE^3^  RE^4^  R^2^ / R^2^ Adjusted | 146.0261***  -2.8136***  0.9096 / 0.9060 | | 132.6094***  0.0806  -0.1347*  0.9222 / 0.9157 | | 159.7092***  -8.647  0.7280  -0.0266  0.9276/ 0.9182 | | 199.63**  -26.2177  3.4085  -0.1987  0.0039  0.9287/ 0.9157 |
| Denmark | Intercept  RE  RE^2^  RE^3^  RE^4^  R^2^ / R^2^ Adjusted | 71.9942***  -1.1179***  0.9328 / 0.9301 | | 74.0109***  -1.3485***  0.0051  0.9339 / 0.9284 | | 80.0655***  -2.4376*  0.0605  -0.0008  0.9359/ 0.9276 | | 103.0544***  -7.9679  0.5027  -0.0150  0.0002  0.9400 / 0.9291 |
| Estonia | Intercept  RE  RE^2^  RE^3^  RE^4^  R^2^ / R^2^ Adjusted | 21.7651***  -0.2204**  0.1573 / 0.1236 | | 1.9976  1.5884*  -0.0397**  0.2893 / 0.2300 | | 68.8624**  -8.3609**  0.4379**  -0.0073**  0.4582/ 0.3875 | | 107.4222  -16.2531  1.0120  -0.0253  0.0002  0.4612 / 0.3632 |
| Finland | Intercept  RE  RE^2^  RE^3^  RE^4^  R^2^ / R^2^ Adjusted | 104.5182***  -1.3821***  0.8142 / 0.8068 | | 41.4904  2.1661  -0.0484  0.8323 / 0.8184 | | -176.8604  18.4695  -0.5082  0.0042  0.8361/ 0.8147 | | -894.7903  103.1486  -4.0689  0.0700  -0.0005  0.8383 / 0.8089 |
| France | Intercept  RE  RE^2^  RE^3^  RE^4^  R^2^ / R^2^ Adjusted | 559.7212***  -16.4951***  0.8689 / 0.8637 | | 551.8368***  -15.1102  -0.0585  0.8690 / 0.8580 | | -360.7272  224.3484  -20.5986  0.5763  0.8811 / 0.8656 | | 1339.809  -368.956  55.8876  -3.7421  0.0901  0.8819 / 0.8605 |
| Germany | Intercept  RE  RE^2^  RE^3^  RE^4^  R^2^ / R^2^ Adjusted | 974.8175***  -14.0065***  0.8789 / 0.8741 | | 924.4883***  0.6342  -0.7511***  0.9210 / 0.9144 | | 1016.802***  -40.8302***  3.9046***  -0.1495***  0.9508 / 0.9444 | | 1001.019***  -31.3468  2.1358  -0.02276  -0.0030  0.9511/ 0.9422 |
| Greece | Intercept  RE  RE^2^  RE^3^  RE^4^  R^2^ / R^2^ Adjusted | 132.5944***  -3.5665***  0.7876 / 0.7791 | | 84.5844***  5.1393  -0.3456*  0.8151 / 0.7997 | | 104.6485  0.5208  0.0367  -0.0100  0.8154/ 0.7913 | | 131.8525  8.7948  -1.0033  0.0458  -0.0010  0.8154/ 0.7818 |
| Hungary | Intercept  RE  RE^2^  RE^3^  RE^4^  R^2^ / R^2^ Adjusted | 67.7622***  -1.2780***  0.9295 / 0.9267 | | 66.3866***  -0.9544  -0.0151  0.9305 / 0.9247 | | 45.3450***  6.0479**  -0.6951***  0.0202***  0.9523 / 0.9461 | | 68.8005**  -4.1725  0.8568  -0.07771  0.0021  0.9540/ 0.9457 |
| Iceland | Intercept  RE  RE^2^  RE^3^  RE^4^  R^2^ / R^2^ Adjusted | 0.8667***  0.0337***  0.7591 / 0.7495 | | -8.1944***  0.3008***  -0.0019***  0.9083 / 0.9007 | | 12.8952  -0.6243*  0.0115**  -0.0001***  0.9322/ 0.9234 | | 203.7662***  -11.9752***  0.2618***  -0.0025***  0.0000**  0.9491 / 0.9399 |
| Ireland | Intercept  RE  RE^2^  RE^3^  RE^4^  R^2^ / R^2^ Adjusted | 45.3817***  -0.7275***  0.3586 / 0.3330 | | 44.4397***  -0.2904  -0.0345  0.3635 / 0.3104 | | 33.011***  7.4426**  -1.3686**  0.0658**  0.4916/ 0.4253 | | 2.2070  33.9777***  -8.5386***  0.8202***  -0.0270***  0.6590 / 0.5970 |
| Italy | Intercept  RE  RE^2^  RE^3^  RE^4^  R^2^ / R^2^ Adjusted | 537.7056***  -10.4697***  0.8095 / 0.8019 | | 412.5909***  17.9807***  -1.2618***  0.9266 / 0.9205 | | 176.3227***  95.0584***  -8.4978***  0.2053***  0.9737 / 0.9703 | | 4.6447  166.3127***  -18.9067***  0.8280**  -0.0130*  0.9771/ 0.9729 |
| Japan | Intercept  RE  RE^2^  RE^3^  RE^4^  R^2^ / R^2^ Adjusted | 1426.0320***  -40.1688***  0.5013 / 0.4814 | | 1026.2950***  115.2393  -14.2108*  0.5587 / 0.5220 | | 93.6381  656.062  -115.6331  6.1520  0.5818/ 0.5273 | | 7609.199  -5151.899  1526.349  -195.0375  9.0258  0.6141 / 0.5439 |
| Latvia | Intercept  RE  RE^2^  RE^3^  RE^4^  R^2^ / R^2^ Adjusted | 13.1700***  -0.1457***  0.5770 / 0.5600 | | 59.7466***  -2.6707***  0.0340***  0.7887 / 0.7711 | | 277.0622**  -20.3953**  0.5139*  -0.0043*  0.8178 / 0.7940 | | 646.8898  -60.3852  2.1304  -0.0333  0.0002  0.8182 / 0.7852 |
| Liechtenstein | Intercept  RE  RE^2^  RE^3^  RE^4^  R^2^ / R^2^ Adjusted | 0.7422***  -0.0103***  0.7470 / 0.7369 | | -4.7715***  0.1981***  -0.0020***  0.8335 / 0.8196 | | -52.2017  2.8806  -0.0525  0.0003  0.8440 / 0.8236 | | -13.8391  0  0.0286  -0.0007  0.0000  0.8436 / 0.8232 |
| Lithuania | Intercept  RE  RE^2^  RE^3^  RE^4^  R^2^ / R^2^ Adjusted | 15.0081***  -0.0497*  0.1308 / 0.0960 | | 19.3419***  -0.4679**  0.0090**  0.3061 / 0.2483 | | 23.8036***  -1.1681  0.0431  -0.0005  0.3269/ 0.2391 | | 34.0696  -3.3428  0.2053  -0.0056  0.0001  0.3329 / 0.2116 |
| Luxembourg | Intercept  RE  RE^2^  RE^3^  RE^4^  R^2^ / R^2^ Adjusted | 10.7244***  -0.1327***  0.2441 / 0.2139 | | 12.2696***  -0.6873***  0.0321***  0.4706 / 0.4265 | | 11.8600***  -0.4426  -0.0041  0.0014  0.4782/ 0.4101 | | 10.9481***  0.3726  -0.2108  0.0202  -0.0005  0.4934 / 0.4013 |
| Macao | Intercept  RE  RE^2^  RE^3^  RE^4^  R^2^ / R^2^ Adjusted | 1.6108***  -0.0232**  0.1703 / 0.1372 | | 1.5777***  -0.0036  -0.0015  0.1862 / 0.1184 | | 1.4984***  0.0846  -0.0170  0.0006  0.2596/ 0.1630 | | 1.4196***  0.2549*  -0.0751*  0.0068  -0.0002  0.3213 / 0.1979 |
| Malta | Intercept  RE  RE^2^  RE^3^  RE^4^  R^2^ / R^2^ Adjusted | 2.6908***  -0.1414***  0.8815 / 0.8767 | | 2.6533***  -0.0432  -0.0129**  0.9072 / 0.8995 | | 2.6207***  0.1513**  -0.0768***  0.0050***  0.9342 / 0.9256 | | 2.6171***  0.1929  -0.1014  0.0092  -0.0002  0.9351/ 0.9233 |
| Netherlands | Intercept  RE  RE^2^  RE^3^  RE^4^  R^2^ / R^2^ Adjusted | 184.6319***  -4.1586***  0.6876 / 0.6751 | | 176.1866***  0.9385  -0.5757**  0.7381 / 0.7163 | | 154.939***  20.6598**  -5.3846**  0.3383**  0.7910/ 0.7637 | | 181.3018***  -10.9158  6.5942  -1.4584  0.0924  0.8073 / 0.7722 |
| New Zealand | Intercept  RE  RE^2^  RE^3^  RE^4^  R^2^ / R^2^ Adjusted | 29.7598**  0.1546  0.0067 / -0.0330 | | -18.3130  3.3795  -0.0540  0.0088 / -0.0738 | | 239.6747  -22.6217  0.8181  -0.0097  0.0089 / -0.1203 | | -15799.7  2129.005  -107.2743  2.4005  -0.0201  0.0104 / -0.1695 |
| Norway | Intercept  RE  RE^2^  RE^3^  RE^4^  R^2^ / R^2^ Adjusted | 69.6660***  -0.4484**  0.1697 / 0.1365 | | 625.5773  -19.3432  0.1604  0.2425 / 0.1794 | | -5166.284  274.3962  -4.8024  0.0279  0.2483 / 0.1502 | | -1012.545  0  1.9885  -0.0467  0.0003  0.2476 / 0.1495 |
| Poland | Intercept  RE  RE^2^  RE^3^  RE^4^  R^2^ / R^2^ Adjusted | 360.9289***  -3.4997**  0.2160 / 0.1847 | | 513.0673***  -38.6434**  1.9037**  0.3388 / 0.2837 | | 1481.631***  -375.6195***  39.6631***  -1.3701***  0.6399/ 0.5929 | | 4415.087***  -1745.293***  273.776***  -18.7056***  0.4700**  0.7258 / 0.6760 |
| Portugal | Intercept  RE  RE^2^  RE^3^  RE^4^  R^2^ / R^2^ Adjusted | 108.0090***  -2.1003***  0.8708 / 0.8656 | | 129.5669***  -3.9191  0.0375  0.8741 / 0.8636 | | -226.8262  41.6206*  -1.8728*  0.0263*  0.8914 / 0.8773 | | 133.4791  -19.4009  1.9600  -0.0795  0.0011  0.8917 / 0.8721 |
| Romania | Intercept  RE  RE^2^  RE^3^  RE^4^  R^2^ / R^2^ Adjusted | 150.7205***  -2.9053***  0.7757 / 0.7668 | | 149.7660***  -2.7955  -0.0030  0.7758 / 0.7571 | | 160.3704  -4.6219  0.1068  -0.0020  0.7760/ 0.7468 | | 374.13441  -60.1906  5.1757  -0.1977  0.0027  0.7859 / 0.7470 |
| Slovenia | Intercept  RE  RE^2^  RE^3^  RE^4^  R^2^ / R^2^ Adjusted | 18.7456***  -0.1756***  0.2473 / 0.2172 | | 3.5108  1.7747***  -0.0585***  0.6926 / 0.6670 | | 4.1466  1.6857  -0.0533  -0.0001  0.6920/ 0.6519 | | 162.183*  -37.1956*  3.4189*  -0.1343*  0.0019*  0.7352 / 0.6870 |
| Spain | Intercept  RE  RE^2^  RE^3^  RE^4^  R^2^ / R^2^ Adjusted | 384.8757***  -7.5459***  0.5440 / 0.5257 | | 358.1763***  -2.8553  -0.1846  0.5453 / 0.5074 | | 900.7255**  -142.6443  11.2374  -0.2979  0.5852 / 0.5311 | | 818.6815  -113.7808  7.5662  -0.0980  -0.0039  0.5853 / 0.5099 |
| Sweden | Intercept  RE  RE^2^  RE^3^  RE^4^  R^2^ / R^2^ Adjusted | 93.7296***  -1.0025***  0.9508 / 0.9488 | | 63.1729***  0.4438  -0.0167**  0.9589 / 0.9555 | | 259.7619***  -13.5714**  0.3114**  -0.0025**  0.9665 / 0.9621 | | 464.2695  -33.2700  1.0148  -0.0136  0.0001  0.9667 / 0.9606 |
| Switzerland | Intercept  RE  RE^2^  RE^3^  RE^4^  R^2^ / R^2^ Adjusted | 65.2198***  -1.1310***  0.7925 / 0.7842 | | -13.8437  6.4621**  -0.1789***  0.8545/ 0.8424 | | -238.2987  38.9838  -1.7377  0.0247  0.8591 / 0.8407 | | 1594.275  -316.7024  24.0125  -0.7994  0.0098  0.8625 / 0.8375 |
| United Kingdom | Intercept  RE  RE^2^  RE^3^  RE^4^  R^2^ / R^2^ Adjusted | 590.7326***  -21.3813***  0.9532 / 0.9514 | | 599.2145***  -27.3342***  0.5237  0.9570/ 0.9535 | | 585.4759***  -12.8303  -2.4694  0.1631  0.9608 / 0.9557 | | 613.7188  -51.1348*  10.5796  -1.3972  0.0604  0.9641/ 0.9576 |
| United States | Intercept  RE  RE^2^  RE^3^  RE^4^  R^2^ / R^2^ Adjusted | 6572.887***  -131.1149***  0.5946 / 0.5784 | | 4309.0360***  528.9035***  -44.1824***  0.7416/ 0.7201 | | -1256.223  2947.999**  -378.9743**  14.8302**  0.7897 / 0.7623 | | -24477.2  16417.1**  -3215.973**  272.2552**  -8.5116*  0.8222 / 0.7899 |
| **Developing economies** | | | | | | | | |
| Algeria | Intercept  RE  RE^2^  RE^3^  RE^4^  R^2^ / R^2^ Adjusted | 174.1954***  -169.8875***  0.7737 / 0.7646 | | 193.4056***  -361.3579***  322.4405**  0.8163 / 0.8009 | | 160.5647***  156.6288  -1642.445**  2104.304**  0.8607 / 0.8425 | | 159.7149***  175.8427  -1767.842  2406.611  -241.2679  0.8607 / 0.8354 |
| Argentina | Intercept  RE  RE^2^  RE^3^  RE^4^  R^2^ / R^2^ Adjusted | 317.4909***  -15.5224***  0.5243 / 0.5053 | | -125.8727  76.5728  -4.7230*  0.5896 / 0.5554 | | -379.7778  157.4511  -13.2290  0.2954  0.5899 / 0.5364 | | -13517.38  5724.042  -891.1646  61.3929  -1.5834  0.5963/0.5229 |
| Aruba | Intercept  RE  RE^2^  RE^3^  RE^4^  R^2^ / R^2^ Adjusted | 2.0420***  -0.1292***  0.2640 / 0.2346 | | 1.9201***  0.3579  -0.0653  0.3388 / 0.2837 | | 1.0894***  5.0392**  -1.3619**  0.0909**  0.4877 / 0.4209 | | -0.8130  16.1949**  -6.2209**  0.8107**  -0.0353***  0.5647/0.4856 |
| Bahamas | Intercept  RE  RE^2^  RE^3^  RE^4^  R^2^ / R^2^ Adjusted | 2.3852***  -0.2001**  0.1761 / 0.1432 | | 2.4627***  -0.2958  0.0258  0.1811 / 0.1129 | | 1.2135***  2.9748***  -2.1661***  0.3899***  0.5764 / 0.5212 | | -1.2837**  12.8520***  -13.6421***  5.4437***  -0.7188***  0.8065/0.8065 |
| Barbados | Intercept  RE  RE^2^  RE^3^  RE^4^  R^2^ / R^2^ Adjusted | 1.4273***  -0.0211**  0.2225 / 0.1914 | | 1.0065***  0.1072***  -0.0072***  0.7055 / 0.6809 | | 0.8489***  0.1867**  -0.0172*  0.0004  0.7230 / 0.6868 | | 0.9077***  0.1409  -0.0066  -0.0006  0.0000  0.7246 / 0.6745 |
| Belize | Intercept  RE  RE^2^  RE^3^  RE^4^  R^2^ / R^2^ Adjusted | 0.6490***  -0.0046  0.0431 / 0.0049 | | 1.1969  -0.0340  0.0004  0.0570 / -0.0216 | | 1.8026  -0.0826  0.0017  -0.0000  0.0573 / -0.0656 | | 32.5989  -3.3501  0.1301  -0.0022  0.0001  0.0677/ -0.1018 |
| Bolivia | Intercept  RE  RE^2^  RE^3^  RE^4^  R^2^ / R^2^ Adjusted | 24.5441***  -0.4867***  0.8350 / 0.8285 | | 28.9816***  -1.1310***  0.0169***  0.8950 / 0.8863 | | 21.728***  0.7403**  -0.1055***  0.0022***  0.9701 / 0.9662 | | 20.5045***  1.2336*  -0.1620**  0.0046*  -0.0000  0.9712 / 0.9660 |
| Botswana | Intercept  RE  RE^2^  RE^3^  RE^4^  R^2^ / R^2^ Adjusted | 9.9686***  -0.1663***  0.7013 / 0.6894 | | 15.7718***  -0.5287**  0.0054*  0.7406 / 0.7189 | | 7.1039  0.2965  -0.0199  0.0003  0.7462 / 0.7131 | | -95.0374  13.6611*  -0.6576*  0.0134*  -0.0001*  0.7768 / 0.7362 |
| Brazil | Intercept  RE  RE^2^  RE^3^  RE^4^  R^2^ / R^2^ Adjusted | 453.0028  -0.9547  0.0005 / -0.0395 | | 5761.212  -237.8547  2.6385  0.0176 / -0.0643 | | 111387.9  -7286.316  159.2055  -1.1577  0.0345 / -0.0914 | | 30516. 54  0  -86.6637  2.525  -0.02066  0.0360/ -0.0898 |
| Cape Verde | Intercept  RE  RE^2^  RE^3^  RE^4^  R^2^ / R^2^ Adjusted | 1.0573***  -0.0234***  0.8421 / 0.8358 | | 1.7175***  -0.0712***  0.0008**  0.8721 / 0.8614 | | 1.1901  -0.0122  -0.0013  0.0000  0.8728 / 0.8562 | | -0.6245  0.2533  -0.0156  0.0004  -0.0000  0.8731 / 0.8501 |
| China | Intercept  RE  RE^2^  RE^3^  RE^4^  R^2^ / R^2^ Adjusted | 13449.12***  -341.6103***  0.9021 / 0.8982 | | 15422.9***  -578.3475***  5.9148  0.9106 / 0.9032 | | 10979.09***  364.8231  -53.5310*  1.1049*  0.9231 / 0.9131 | | 2766.797  2806.673  -294.7265  10.6767  -0.1314  0.9290 / 0.9161 |
| Coasta Rica | Intercept  RE  RE^2^  RE^3^  RE^4^  R^2^ / R^2^ Adjusted | -0.3037  0.1947***  0.3726 / 0.3475 | | -19.9185  1.2961  -0.0153  0.4069 / 0.3574 | | 195.7085  -17.0727  0.5022  -0.0048  0.4538 / 0.3825 | | 339.1992  -33.4215  1.1967  -0.0179  0.0001  0.4539 / 0.3546 |
| Colombia | Intercept  RE  RE^2^  RE^3^  RE^4^  R^2^ / R^2^ Adjusted | -17.2628  2.9736**  0.1534 / 0.1195 | | -432.686  30.9395  -0.4686  0.1802 / 0.1118 | | 20484.15***  -2092.611***  71.0886***  -0.8004***  0.5288 / 0.4673 | | -96786.75  13739.15  -727.9844  17.0717  -0.1494  0.5653/ 0.4863 |
| Curacao | Intercept  RE  RE^2^  RE^3^  RE^4^  R^2^ / R^2^ Adjusted | 4.7657***  -0.2659  0.0207 / -0.0185 | | 3.5740***  3.2224**  -1.2912***  0.2933 / 0.2344 | | 2.6598***  7.7382***  -5.4135***  0.9549**  0.4259 / 0.3510 | | 3.3468***  0.7731  8.0050  -6.8439  1.36845  0.5419/ 0.4586 |
| Dominican Republic | Intercept  RE  RE^2^  RE^3^  RE^4^  R^2^ / R^2^ Adjusted | 49.1089***  -1.5917***  0.8237 / 0.8167 | | 90.6385***  -6.3067***  0.1322**  0.8589 / 0.8471 | | 200.6408  -24.9231  1.1711  -0.0191  0.8630 / 0.8451 | | -55.0262  32.7074  -3.6605  0.1594  -0.0025  0.8633 / 0.8384 |
| Ecuador | Intercept  RE  RE^2^  RE^3^  RE^4^  R^2^ / R^2^ Adjusted | 63.6704***  -2.0370***  0.4953 / 0.4752 | | 60.1131  -1.5796  -0.0143  0.4955 / 0.4535 | | 15.2329  6.9601  -0.5456  0.0108  0.4960 / 0.4303 | | -58.0094  25.6866  -2.3187  0.0845  -0.0011  0.4960 / 0.4044 |
| Egypt | Intercept  RE  RE^2^  RE^3^  RE^4^  R^2^ / R^2^ Adjusted | 415.5676***  -35.9881***  0.9210 / 0.9179 | | 444.4589***  -44.971**  0.6677  0.9215 / 0.9149 | | -115.6387  226.2235  -41.9822*  2.1765*  0.9307 / 0.9217 | | -5179.348***  3510.775***  -822.176***  82.6706***  -3.0483***  0.9534/ 0.9449 |
| El Salvador | Intercept  RE  RE^2^  RE^3^  RE^4^  R^2^ / R^2^ Adjusted | 7.6684***  -0.0451***  0.6901 / 0.6778 | | 7.0747***  -0.0089  -0.0005  0.7029 / 0.6781 | | 11.9649***  -0.5113**  0.0152**  -0.0001**  0.7727 / 0.7431 | | 8.1888*  -0.0040  -0.0087  0.0003  -3.35  0.7806/ 0.7408 |
| Eswatini | Intercept  RE  RE^2^  RE^3^  RE^4^  R^2^ / R^2^ Adjusted | 1.1016***  -0.0020  0.0057 / -0.0341 | | 1.0951*  -0.0015  -0.0000  0.0057 / -0.0772 | | 0.2581  0.1239  -0.0060  0.0001  0.0169 / -0.1113 | | 10.4789  -1.9508  0.1453  -0.0046  0.0001  0.0667/-0.1030 |
| Fiji | Intercept  RE  RE^2^  RE^3^  RE^4^  R^2^ / R^2^ Adjusted | 1.7219***  -0.1774***  0.7441 / 0.7338 | | 2.2788***  -0.0473***  0.0004*  0.7827 / 0.7646 | | 2.0858*  -0.0313  -0.0001  0.0000  0.7830 / 0.7547 | | 4.8801  -0.3439  0.0125  -0.0002  0.0000  0.7894 / 0.7511 |
| French Polynesia | Intercept  RE  RE^2^  RE^3^  RE^4^  R^2^ / R^2^ Adjusted | 1.1637***  -0.0497**  0.1588 / 0.1251 | | 2.2751  -0.3079  0.0146  0.1813 / 0.1131 | | 2.6917  -0.4555  0.0318  -0.0007  0.1814 / 0.0746 | | -149.4706**  71.1089*  -12.4379*  0.9535*  -0.0271*  0.3340/ 0.2130 |
| Ghana | Intercept  RE  RE^2^  RE^3^  RE^4^  R^2^ / R^2^ Adjusted | 29.6843***  -0.3321***  0.8443 / 0.8381 | | 61.2932***  -1.4797***  0.0099***  0.9512 / 0.9471 | | 65.5693***  -1.7185  0.0142  -0.0000  0.9513 / 0.9449 | | -135.2433  13.53396*  -.4082793*  .005036*  -.0000222*  0.9623/ 0.9555 |
| Guatemala | Intercept  RE  RE^2^  RE^3^  RE^4^  R^2^ / R^2^ Adjusted | 25.7118  -0.2040  0.0076 / -0.0321 | | -944.0291  29.8071  -0.2320  0.0402 / -0.0398 | | -47787.96  2208.868  -34.0039  0.1744  0.0904 / -0.0282 | | -12254.49  0  17.4689  -0.3585  0.0021  0.0904 / -0.0282 |
| Guyana | Intercept  RE  RE^2^  RE^3^  RE^4^  R^2^ / R^2^ Adjusted | 3.4793***  -0.0505***  0.7076 / 0.6959 | | 3.9838***  -0.0919*  0.0008  0.7170 / 0.6934 | | 1.2377  0.2787  -0.0146*  0.0002*  0.7600 / 0.7287 | | -9.6247**  2.3241  -0.1487  0.0038  -0.0001  0.8157/ 0.7822 |
| Honduras | Intercept  RE  RE^2^  RE^3^  RE^4^  R^2^ / R^2^ Adjusted | 24.1448***  -0.3135***  0.5280 / 0.5092 | | 18.8374  -0.1194  -0.0017  0.5286 / 0.4893 | | 57.9548  -2.3085  0.0388  -0.0002  0.5288 / 0.4673 | | 5681.007  -413.8172  11.273  -0.1359  0.0006  0.5671/ 0.4884 |
| Hong Kong | Intercept  RE  RE^2^  RE^3^  RE^4^  R^2^ / R^2^ Adjusted | 42.4062***  -9.4304***  0.2573 / 0.2276 | | 36.5090***  45.4867***  -63.0845***  0.7337 / 0.7115 | | 34.8195***  70.3927***  -140.9805**  60.7961  0.7538 / 0.7217 | | 30.9763***  158.1091**  -660.0957**  1074.19*  -610.0015*  0.7873/ 0.7486 |
| India | Intercept  RE  RE^2^  RE^3^  RE^4^  R^2^ / R^2^ Adjusted | 4699.446***  -76.1397***  0.9006 / 0.8966 | | 8419.267***  -262.2506***  2.2395***  0.9390 / 0.9340 | | -1452.636  490.2263  -16.4380**  0.1510**  0.9501 / 0.9436 | | -79924.69***  8699.888***  -332.3255***  5.4499***  -0.0327**  0.9656 / 0.9594 |
| Indonesia | Intercept  RE  RE^2^  RE^3^  RE^4^  R^2^ / R^2^ Adjusted | 911.4116***  -13.7174***  0.9789 / 0.9780 | | 833.0899***  -8.8082**  -0.0708  0.9804 / 0.9787 | | 945.2077***  -19.5220  0.2531  -0.0031  0.9806 / 0.9781 | | 2435.273**  -210.7437  9.0919  -0.1780  0.0013  0.9821 / 0.9789 |
| Iran | Intercept  RE  RE^2^  RE^3^  RE^4^  R^2^ / R^2^ Adjusted | 348.4317***  180.8861  0.0780 / 0.0412 | | -150.1831  1314.209*  -606.5885*  0.1792 / 0.1108 | | 902.412  -2555.799  3789.088  -1559.837  0.2101 / 0.1071 | | 1363.496  -4914.444  8077.658  -4862.236  911.2498  0.2106 / 0.0671 |
| Iraq | Intercept  RE  RE^2^  RE^3^  RE^4^  R^2^ / R^2^ Adjusted | 117.9962***  5.8373  0.0059 / -0.0338 | | 61.9917**  138.0502**  -52.3963**  0.2362 / 0.1725 | | -92.3747*  711.667***  -569.9013***  126.6604***  0.4773 / 0.4091 | | -298.8446**  1663.337**  -1884.858**  825.4303**  -124.7622*  0.5484 / 0.4663 |
| Israel | Intercept  RE  RE^2^  RE^3^  RE^4^  R^2^ / R^2^ Adjusted | 56.7788***  0.6262  0.0397 / 0.0013 | | 98.9496***  -14.7972***  1.2906***  0.6125 / 0.5802 | | 191.5657***  -66.8731***  10.3977***  -0.4999***  0.7802 / 0.7516 | | 227.2287***  -94.8101*  18.2289  -1.4323  0.0399  0.7830 / 0.7435 |
| Jordan | Intercept  RE  RE^2^  RE^3^  RE^4^  R^2^ / R^2^ Adjusted | 14.3407***  1.8645***  0.4869 / 0.4664 | | 4.9005  7.1425***  -0.5791***  0.6301 / 0.5992 | | -5.9515  15.8823**  -2.6301  0.1426  0.6544 / 0.6093 | | 18.7761  -11.606  7.8635  -1.4815  0.0867  0.6760/ 0.6171 |
| Kenya | Intercept  RE  RE^2^  RE^3^  RE^4^  R^2^ / R^2^ Adjusted | 86.7648***  -0.9674***  0.9171 / 0.9138 | | 17.4715  0.8642  -0.0121  0.9190 / 0.9122 | | -2618.637*  105.3613*  -1.4903*  0.0060*  0.9314 / 0.9225 | | -6547.908  314.1115  -5.5421  0.04268  -0.000  0.9315 / 0.9190 |
| Lebanon | Intercept  RE  RE^2^  RE^3^  RE^4^  R^2^ / R^2^ Adjusted | 29.3324***  -1.7953**  0.1666 / 0.1332 | | 34.6687  -3.7248  0.1678  0.1685 / 0.0992 | | 207.5262  -97.4472  16.6738  -0.9441  0.2364 / 0.1368 | | 1387.943**  -943.596***  239.854**  -26.62273**  1.088049**  0.3424 / 0.2228 |
| Malaysia | Intercept  RE  RE^2^  RE^3^  RE^4^  R^2^ / R^2^ Adjusted | 257.5986***  -16.0677***  0.3124 / 0.2849 | | 210.2843***  6.334  -2.1941  0.3319 / 0.2762 | | 227.2338  -5.5163  0.2679  -0.1545  0.3323 / 0.2452 | | 1051.525**  -780.193**  250.3717**  -33.13708**  1.51719**  0.4647 / 0.3674 |
| Maldives | Intercept  RE  RE^2^  RE^3^  RE^4^  R^2^ / R^2^ Adjusted | 2.1286***  -0.6822***  0.7511 / 0.7412 | | 3.5572***  -2.3469***  0.4163***  0.9099 / 0.9024 | | 3.8326***  -2.8753***  0.7185  -0.0520  0.9113 / 0.8998 | | 1.7811  2.8561  -4.643807**  1.971237**  -.2638427**  0.9252 / 0.9116 |
| Mexico | Intercept  RE  RE^2^  RE^3^  RE^4^  R^2^ / R^2^ Adjusted | 709.8339***  -25.9936***  0.5844 / 0.5677 | | -619.1534*  222.9645***  -11.4317***  0.7607 / 0.7408 | | -7400.504***  2169.097***  -195.5829***  5.7408***  0.8335 / 0.8117 | | -63534.78***  23524.11***  -3216.94***  194.1586***  -4.370441***  0.9165/0.9014 |
| Micronesia | Intercept  RE  RE^2^  RE^3^  RE^4^  R^2^ / R^2^ Adjusted | 0.1781***  -0.0206  0.0304 / -0.0084 | | 0.0726  0.1338  -0.0550  0.0594 / -0.0190 | | 0.7694  -1.4529  1.1069  -0.2749  0.1242 / 0.0099 | | -0.8072  3.386685  -4.310608  2.353436  -.4671677  0.1384/0.0183 |
| Mongolia | Intercept  RE  RE^2^  RE^3^  RE^4^  R^2^ / R^2^ Adjusted | 55.0287***  -8.3035***  0.3416 / 0.3152 | | 125.0086**  -40.7631  3.527  0.3880 / 0.3370 | | 99.8254  -23.3683  -0.3459  0.2781  0.3882 / 0.3084 | | -450.5762  481.462  -169.9005  24.99397  -1.320679  0.3951/0.2851 |
| Morocco | Intercept  RE  RE^2^  RE^3^  RE^4^  R^2^ / R^2^ Adjusted | 89.6625***  -2.8883***  0.5711 / 0.5540 | | 189.7972***  -16.7182***  0.4528***  0.7886 / 0.7710 | | 198.1593  -18.3874  0.5592  -0.0022  0.7887 / 0.7611 | | -423.3787  146.2544  -15.37025  .665473  -.0102317  0.7993/0.7628 |
| Namibia | Intercept  RE  RE^2^  RE^3^  RE^4^  R^2^ / R^2^ Adjusted | 14.2041***  -0.3554***  0.6316 / 0.6168 | | 81.3056**  -4.5162**  0.0642**  0.6886 / 0.6626 | | -607.2644  59.4150  -1.9099  0.0203  0.7102 / 0.6724 | | -1255.027  139.8907  -5.6526  0.0975  -0.0006  0.7103 / 0.6576 |
| New Caledonia | Intercept  RE  RE^2^  RE^3^  RE^4^  R^2^ / R^2^ Adjusted | 6.7965***  -0.5828***  0.3612 / 0.3357 | | 8.7026*  -1.2240  0.0512  0.3667 / 0.3139 | | 5.0419  0.6184  -0.2477  0.0156  0.3681 / 0.2857 | | 39.4591  -23.1315  5.717694  -.6306635  0.0255136  0.3754/0.2618 |
| Nicaragua | Intercept  RE  RE^2^  RE^3^  RE^4^  R^2^ / R^2^ Adjusted | 12.5146***  -0.1503***  0.8567 / 0.8510 | | 6.7950  0.0550  -0.0018  0.8598 / 0.8481 | | -80.7513  4.8108  -0.0875  0.0005  0.8642 / 0.8465 | | -1933.594  138.5768  -3.696149  .0436251  -.0001925  0.8740/0.8511 |
| Nigeria | Intercept  RE  RE^2^  RE^3^  RE^4^  R^2^ / R^2^ Adjusted | 954.5183***  -10.1798***  0.5696 / 0.5524 | | -2205.661  64.6078  -0.4422  0.5738 / 0.5383 | | -139603  4934.086  -57.9438  0.2262  0.5795 / 0.5247 | | -36635.46  0  30.67291  -.4807494  0.0021141  0.5800/0.5252 |
| Pakistan | Intercept  RE  RE^2^  RE^3^  RE^4^  R^2^ / R^2^ Adjusted | 736.1337***  -12.3730***  0.9514 / 0.9495 | | 1622.122***  -49.8283***  0.3940**  0.9604 / 0.9571 | | 8406.33  -481.4148  9.5188  -0.06412  0.9618 / 0.9568 | | 51463.23  -4110.489  123.9967  -1.6660  0.0084  0.9621 / 0.9552 |
| Panama | Intercept  RE  RE^2^  RE^3^  RE^4^  R^2^ / R^2^ Adjusted | 18.0311***  -0.3927***  0.7212 / 0.7100 | | 30.6724***  -1.3733***  0.0180**  0.7702 / 0.7511 | | 31.1753  -1.4337  0.0203  -0.0000  0.7702 / 0.7402 | | 12.1903  1.6460  -0.1622  0.0047  -0.000  0.7705 / 0.7287 |
| Paraguay | Intercept  RE  RE^2^  RE^3^  RE^4^  R^2^ / R^2^ Adjusted | 31.2546***  -0.4002***  0.8101 / 0.8025 | | 236.7326***  -6.7578***  0.0490***  0.9265 / 0.9204 | | 1184.546  -50.6990  0.7268  -0.0035  0.9318 / 0.9229 | | 364.9267*  0  -0.4477*  0.0085  -0.0000  0.9318 / 0.9229 |
| Peru | Intercept  RE  RE^2^  RE^3^  RE^4^  R^2^ / R^2^ Adjusted | 99.9709***  -1.8318***  0.5181 / 0.4988 | | 259.1529**  -11.4436*  0.1422  0.5624 / 0.5259 | | 1428.56  -117.6527  3.3235  -0.0314  0.5874 / 0.5336 | | 4918.849  -533.7437  21.7862  -0.3929  0.0026  0.5894 / 0.5147 |
| Philippines | Intercept  RE  RE^2^  RE^3^  RE^4^  R^2^ / R^2^ Adjusted | 346.7709***  -8.0172***  0.6465 / 0.6324 | | 1194.818***  -61.2101***  0.8278***  0.7530 / 0.7324 | | 4795.683  -395.0016  11.0613  -0.1038  0.7678 / 0.7375 | | -19641.76  2649.429  -130.4284  2.803374  -.0222797  0.7741/0.7330 |
| Saudi Arabia | Intercept  RE  RE^2^  RE^3^  RE^4^  R^2^ / R^2^ Adjusted | 333.5483***  9695.746*  0.1050 / 0.0692 | | -176.3411  79038*  -1921752*  0.2124 / 0.1468 | | -3950.481***  790330.3***  -0.0000***  -0.0000***  0.4925 / 0.4263 | | -3072.499  577108.6  -2.40e+07  4.32e+07  8.42e+09  0.4936/0.4016 |
| Seychelles | Intercept  RE  RE^2^  RE^3^  RE^4^  R^2^ / R^2^ Adjusted | 0.5244***  -0.0929**  0.1613 / 0.1277 | | 0.3066*  0.2069  -0.0863  0.2354 / 0.1717 | | -0.0341  0.9371  -0.5595  0.0907  0.2563 / 0.1593 | | 1.3166  -2.826269  3.092623  -1.370189  0.2043166  0.2853/0.1554 |
| Singapore | Intercept  RE  RE^2^  RE^3^  RE^4^  R^2^ / R^2^ Adjusted | 64.3553***  -34.2403**  0.2213 / 0.1902 | | 23.1890  113.1349  -126.7469  0.2870 / 0.2276 | | 31.8115  65.6476  -42.5929  -48.0164  0.2873 / 0.1943 | | 437.1859  -3013.257  8421.943  -10050.53  4295.554  0.3177/0.1937 |
| South Africa | Intercept  RE  RE^2^  RE^3^  RE^4^  R^2^ / R^2^ Adjusted | 562.4024***  -10.8262***  0.6484 / 0.6343 | | 390.2147***  16.3486  -1.0096  0.6859 / 0.6597 | | -628.9526*  274.2466***  -22.0158***  0.5485***  0.7816 / 0.7532 | | -1043.57  414.2771  -39.20423  1.458473  -.0175695  0.7823/0.7427 |
| Sri Lanka | Intercept  RE  RE^2^  RE^3^  RE^4^  R^2^ / R^2^ Adjusted | 68.8589***  -0.9227***  0.9350 / 0.9324 | | 69.4279**  -0.9425  0.0002  0.9350 / 0.9296 | | -557.1872  30.8739  -0.5344  0.0030  0.9423 / 0.9348 | | 3079.767  -216.1747  5.7293  -0.0673  0.0003  0.9449 / 0.9348 |
| Suriname | Intercept  RE  RE^2^  RE^3^  RE^4^  R^2^ / R^2^ Adjusted | 2.9217***  -0.0363***  0.3162 / 0.2889 | | 4.2354***  -0.1778*  0.0035  0.3829 / 0.3314 | | 2.1554  0.1577  -0.0135  0.0003  0.3926 / 0.3134 | | -4.0243  1.4961  -0.1179  0.0038  -0.0000  0.3978 / 0.2883 |
| Syria | Intercept  RE  RE^2^  RE^3^  RE^4^  R^2^ / R^2^ Adjusted | 39.4556***  3.0669  0.0198 / -0.0194 | | 4.6752  47.2039**  -12.4731**  0.2205 / 0.1555 | | 8.3717  39.5595  -7.8077  -0.8568  0.2209 / 0.1192 | | 36.7064  -42.0516  71.8812  -32.6677  4.4273  0.2261 / 0.0854 |
| Thailand | Intercept  RE  RE^2^  RE^3^  RE^4^  R^2^ / R^2^ Adjusted | -258.6037**  22.3899***  0.4264 / 0.4035 | | 2888.863  -265.7669  6.5699  0.4827 / 0.4395 | | 61710.49*  -8306.062  372.0257  -5.5240  0.5379 / 0.4776 | | -201343.5  39457.7  -2874.711  92.3990  -1.1057  0.5406 / 0.4571 |
| Tunisia | Intercept  RE  RE^2^  RE^3^  RE^4^  R^2^ / R^2^ Adjusted | 72.9996***  -3.5973***  0.6354 / 0.6208 | | 314.6543***  -39.4103***  1.3187***  0.7520 / 0.7313 | | -2003.295**  462.7424***  -34.7504***  0.8592***  0.8261 / 0.8035 | | -1322.738  263.3576  -12.9307  -0.1978  0.0191  0.8262 / 0.7945 |
| Turkey | Intercept  RE  RE^2^  RE^3^  RE^4^  R^2^ / R^2^ Adjusted | 617.8356***  -20.5526***  0.8062 / 0.7984 | | 936.6481***  -62.1393***  1.2844**  0.8410 / 0.8277 | | 164.0523  94.9096  -9.0089  0.2172  0.8515 / 0.8321 | | -773.3302  353.5865  -35.0848  1.3558  -0.0182  0.8521 / 0.8252 |
| Venezuela | Intercept  RE  RE^2^  RE^3^  RE^4^  R^2^ / R^2^ Adjusted | 235.5753**  -5.7351  0.0378 / -0.0007 | | 909.9447  -99.7044  3.2540  0.0608 / -0.0175 | | -7365.069  1645.031  -118.7344  2.8286  0.0917 / -0.0268 | | 36110.1  -10644.39  1178.607  -57.7923  1.0580  0.0973 / -0.0668 |
| Vietnam | Intercept  RE  RE^2^  RE^3^  RE^4^  R^2^ / R^2^ Adjusted | 394.209***  -6.1443***  0.9038 / 0.8999 | | 573.8022***  -16.1352***  0.1217***  0.9694 / 0.9669 | | 674.6682***  -25.1265***  0.3620*  -0.0020  0.9713 / 0.9675 | | 780.0009**  -37.3017  0.8512  -0.0102  0.0000  0.9715 / 0.9663 |
| Zimbabwe | Intercept  RE  RE^2^  RE^3^  RE^4^  R^2^ / R^2^ Adjusted | 33.0198***  -0.2789***  0.7119 / 0.7003 | | 111.2213***  -2.3825**  0.0140**  0.7590 / 0.7389 | | -230.2806  11.241  -0.1659  0.0008  0.7648 / 0.7341 | | 5756.802  -309.7345  6.264695  -.0562798  0.0001893  0.7767/0.7361 |
| **Economies in transition** | | | | | | | | |
| Albania | Intercept  RE  RE^2^  RE3  RE4  R^2^ / R^2^ Adjusted | 9.6427***  -0.1411***  0.5264 / 0.5075 | | -7.4847  -0.6740**  -0.0094  0.6362 / 0.6059 | | -94.4863**  -0.1602**  0.0011**  -94.4863**  0.7126/ 0.6751 | | -126.4725  10.0400  -0.2672  0.0028  -.42e-06  0.7129/ 0.6606 |
| Azerbaijan | Intercept  RE  RE^2^  RE3  RE4  R^2^ / R^2^ Adjusted | 33.4801***  -0.1609  0.0016 / -0.0383 | | 26.3387***  5.2650*  -0.9767*  0.0493 / -0.0299 | | 41.0140*  5.2079  -0.7106  41.0140  0.0699/ -0.0514 | | 113.9276  -127.7393  71.3003  -16.6825  1.3847  0.1122/ -0.0492 |
| Belarus | Intercept  RE  RE^2^  RE3  RE4  R^2^ / R^2^ Adjusted | 59.1281***  0.1118  0.0041/ -0.0358 | | 69.5966***  -4.1881**  0.3928**  0.1246 / 0.0516 | | 106.0995***  4.4206*  -0.2307*  106.0995***  0.2381/ 0.1387 | | -52.9075  108.6668***  -35.8822***  4.8161***  -0.2253***  0.5511/ 0.4695 |
| Georgia | Intercept  RE  RE^2^  RE3  RE4  R^2^ / R^2^ Adjusted | 16.0485***  -0.2443  0.8566/ 0.8509 | | 29.2970***  -0.9566***  0.0089***  0.9446 / 0.9400 | | 23.0750**  -0.4526  -0.0040  0.0001  0.9452/ 0.9381 | | -55.1478  8.0661  -0.3420*  0.0059*  -0.00003*  0.9525/ 0.9438 |
| Kazakhstan | Intercept  RE  RE^2^  RE3  RE4  R^2^ / R^2^ Adjusted | 307.445***  1.8645***  0.4869 / 0.4664 | | 104.9005  7.1425***  -0.5791***  0.6301 / 0.5992 | | 504.5951*  304.7216  -63.2897  504.5951  0.1455/ 0.0341 | | 2942.527  -6099.706  4993.719  -1754.609  222.079  0.1869/ 0.0391 |
| Kyrgyzstan | Intercept  RE  RE^2^  RE3  RE4  R^2^ / R^2^ Adjusted | 17.8162***  -0.9674***  0.9171 / 0.9138 | | 31.2727**  0.8642  -0.0121  0.9190 / 0.9122 | | -44.3626  6.8662  -0.2767  0.0034  0.6213/ 0.5719 | | -1115.293  162.2364  -8.6487  0.2019  -0.0017  0.6475/ -0.5834 |
| Moldova | Intercept  RE  RE^2^  RE3  RE4  R^2^ / R^2^ Adjusted | 6.0427***  -1.7953**  0.1666 / 0.1332 | | 8.8686***  -3.7248  0.1678  0.1685 / 0.0992 | | 21.4971***  -4.6474***  0.2978***  -0.0054***  0.7018/ 0.6629 | | 27.6459***  -7.6102***  0.7443***  -0.0288***  0.0003***  0.7938/ 0.7563 |
| North Macedonia | Intercept  RE  RE^2^  RE3  RE4  R^2^ / R^2^ Adjusted | 18.7107***  -16.0677***  0.3124 / 0.2849 | | 11.0442  6.334  -2.1941  0.3319 / 0.2762 | | -35.1587  7.8468  -0.4236  0.0070  0.4726/ 0.4038 | | 1226.517*  -262.2327  21.0346  -0.7431  0.0097  0.5404 0.4568 |
| Russia | Intercept  RE  RE^2^  RE3  RE4  R^2^ / R^2^ Adjusted | 2403.675***  -232.9485***  0.4204 / 0.3973 | | 11519.33  -5080.706***  696.9986**  0.5476 / 0.5099 | | 17331.47*  -10665.22  2287.625  -150.9186  0.5960/ 0.5433 | | 24138.4  -10976.9  -779.6133  1038.854  -127.6498  0.5994/ 0.5265 |
| Tajikistan | Intercept  RE  RE^2^  RE3  RE4  R^2^ / R^2^ Adjusted | 19.8427***  -0.2868***  0.8171/ 0.8098 | | 51.7576***  -1.5488  0.0121  0.8727 / 0.8621 | | -34.1051  3.5279  -0.0863  0.0006  0.8801/ 0.8645 | | -564.7948  45.9261  -1.3425  0.0169  -0.0007  0.8855/ 0.8647 |
| Ukraine | Intercept  RE  RE^2^  RE3  RE4  R^2^ / R^2^ Adjusted | 348.3705***  -19.9322  0.7300 / 0.7192 | | 355.216***  -25.2898  0.6967  0.7325/ -0.7102 | | 303.2798***  38.2772  -19.1018  1.7036  0.7720/ 0.7422 | | 302.0104***  40.2475  -20.0281  1.8690  -0.0099  0.7720/ 0.7305 |
| Uzbekistan | Intercept  RE  RE^2^  RE3  RE4  R^2^ / R^2^ Adjusted | 132.8734***  -13.6082***  0.3012 / 0.2733 | | 126.6613***  -2.1539  -4.6617  0.3039 / 0.2459 | | 142.169  -43.2394  29.1436  -8.9386  0.3047/ 0.2140 | | 114.5301  56.2179  -100.9872  64.5570  -15.1278  0.3052/ 0.1788 |
| **Least developed economies** | | | | | | | | |
| Angola | Intercept  RE  RE^2^  RE3  RE4  R^2^ / R^2^ Adjusted | 56.1007***  -0.6152***  0.7550/ 0.7452 | | -47.8321*  2.9104***  -0.0290**  0.8522/ 0.8399 | | -515.2599***  27.0814**  -0.4391**  0.0022**  0.8941/ 0.8803 | | -1758.367*  112.393  -2.6097  0.0265  -0.0001  0.9012/ 0.8832 |
| Bangladesh | Intercept  RE  RE^2^  RE3  RE4  R^2^ / R^2^ Adjusted | 130.5773***  -1.8003***  0.9848/ 0.9842 | | 164.0282***  -3.4931  -0.0195*  0.9979 / 0.9978 | | 168.0556***  -3.8033***  0.0270  -0.0001  0.9980/ 0.9977 | | 124.5847***  0.7132  -0.1414  0.0026  -0.0000  0.9980/ 0.9977 |
| Benin | Intercept  RE  RE^2^  RE3  RE4  R^2^ / R^2^ Adjusted | 11.7466***  -0.1349***  0.8265/ 0.8195 | | 22.5727***  -0.4967  -0.0028  0.9286 / 0.9226 | | 15.6371**  -0.1403  -0.0030  0.0000  0.9327/ 0.9239 | | -52.4777**  4.5843***  -0.1212***  0.0012***  -0.91e-06***  0.9567/ 0.9488 |
| Bhutan | Intercept  RE  RE^2^  RE3  RE4  R^2^ / R^2^ Adjusted | 10.7037***  -0.1123**  0.9541 / 0.9523 | | 35.2036**  -0.6629  0.0031  0.9598 / 0.9564 | | -607.0814**  21.2637**  -0.2462**  0.0010**  0.9676/ 0.9634 | | -280.9778**  5.4518  0.0395  -0.0013  6.82e-06  0.9692/ 0.9636 |
| Burundi | Intercept  RE  RE^2^  RE3  RE4  R^2^ / R^2^ Adjusted | 5.1292***  -0.0517**  0.9440 / 0.9418 | | 9.8183  -0.1674***  0.0006***  0.9453 / 09408 | | -372.125***  12.5378**  -0.1399**  0.0005**  0.9635/ 0.9587 | | -363.6369**  11.9621*  -0.1270  0.0003  3.95e-07  0.9635/ 0.9568 |
| Cambodia | Intercept  RE  RE^2^  RE3  RE4  R^2^ / R^2^ Adjusted | 39.0882***  -0.4665  0.8176 / 0.8103 | | 173.3382***  -4.4563  0.0289  0.9639 / 0.9609 | | 680.711***  -27.4981***  0.3735***  -0.0017***  0.9894/ 0.9880 | | 901.6684  -40.7668  0.6697  -0.0046  0.0000  0.9894/ 0.9875 |
| Chad | Intercept  RE  RE^2^  RE3  RE4  R^2^ / R^2^ Adjusted | 6.3703***  -0.0628***  0.7276/ 0.7167 | | 26.3398***  -0.5308***  0.0027***  0.8064 / 0.0436 | | -198.3647***  7.5158**  -0.0927**  0.0003**  0.8683/ 0.8511 | | -1299.094*  61.1108*  -1.06648  0.0081  -0.0000  0.8810/ 0.8594 |
| Comoros | Intercept  RE  RE^2^  RE3  RE4  R^2^ / R^2^ Adjusted | 0.3580***  -0.0032***  0.0804 / 0.0436 | | -1.2605  0.0512**  -0.0004*  0.1733 / 0.1044 | | -16.3964*  0.7967*  -0.0125  0.000  0.2564/ 0.1594 | | -240.7595***  15.5562***  -0.3740***  0.0039***  -0.0000***  0.5700/ 0.4919 |
| Eritrea | Intercept  RE  RE^2^  RE3  RE4  R^2^ / R^2^ Adjusted | 0.6017***  -0.0029  0.3467 / 0.3205 | | 0.6438***  -0.00341  0.0001  0.4108 / 0.3617 | | 0.5863***  0.0086  -0.0003  3.50e-06  0.4418/ 0.3690 | | 0.3887***  0.0679**  -0.0031**  0.0000**  -3.67e-07**  0.5690/ 0,4906 |
| Ethiopia | Intercept  RE  RE^2^  RE3  RE4  R^2^ / R^2^ Adjusted | 211.0633***  -2.1722***  0.9704/ 0.9693 | | 1228.081***  -24.3704***  0.1196**  0.9827 / 0.9812 | | -9525.577**  323.0489**  -3.6166**  0.0133**  0.9863/ 0.9845 | | -12678.81***  233.1782*  1.4721  -0.0491  0.0002**  0.9889/ 0.9869 |
| Gambia | Intercept  RE  RE^2^  RE3  RE4  R^2^ / R^2^ Adjusted | 1.8247***  -0.2555***  0.7468 / 0.7367 | | 6.3447***  -0.1928***  0.0015  0.7864 / 0.7686 | | -37.9346  2.2155  -0.0418  0.0002  0.7890/ 0.7614 | | -35.3989  -0.0366  0.0002  2.95e-07  0.7890/ 0.7506  0.7890/ 0.7506 |
| Guinea | Intercept  RE  RE^2^  RE3  RE4  R^2^ / R^2^ Adjusted | 14.3861***  -0.1525***  0.8848 / 0.8802 | | 44.4659***  -0.9281  -0.0050  0.9292/ 0.9233 | | -58.1185  3.0559  -0.0463  0.0002  0.9334/ 0.9247 | | -1600.525***  83.4546***  -1.6124***  0.0137***  -0.0000***  0.9519/ 0.9432 |
| Lesotho | Intercept  RE  RE^2^  RE3  RE4  R^2^ / R^2^ Adjusted | 3.9976***  -0.0375**  0.4896 / 0.4691 | | 10.8353**  -0.3271  0.0030  0.5318/ 0.4928 | | -18.9725  01.5539  -0.0361  0.0002  0.5375/ 0.4771 | | -1524.024***  128.7103***  -4.0400***  0.05596***  -0.0002***  0.7055/ 0.6520 |
| Malawi | Intercept  RE  RE^2^  RE3  RE4  R^2^ / R^2^ Adjusted | 7.2739***  -0.0773  0.8915 / 0.08872 | | -5.4319*  0.2527**  -0.0021***  0.8980 / 0.8895 | | -326.2581  12.5585  -0.1593  0.0006  0.9082/ 0.8962 | | -230.2249  2.5603  0.1297  -0.0026  0.0000*  0.9194/ 0.9048 |
| Mali | Intercept  RE  RE^2^  RE3  RE4  R^2^ / R^2^ Adjusted | 21.1775***  -0.2360***  0.7233 / 0.7122 | | 63.6817  -1.2722***  0.0064**  0.7313 / 0.7089 | | -229.9232  9.6126  -0.1281  0.0005  0.7328/ 0.6979 | | 978.9217  -55.4223  1.1703  -0.0109  0.0000  0.7415/ 0.6945 |
| Mauritius | Intercept  RE  RE^2^  RE3  RE4  R^2^ / R^2^ Adjusted | 4.9127***  -0.0859***  0.8852 / 0.8806 | | 5.2364***  -0.1254  -0.0009  0.8965 / 0.8879 | | 4.3074***  0.1010*  -0.0133***  0.0002***  0.9477/ 0.9409 | | 3.6396***  0.3594***  -0.0412***  0.0013***  -0.0000***  0.9695/ 0.9639 |
| Mozambique | Intercept  RE  RE^2^  RE3  RE4  R^2^ / R^2^ Adjusted | 36.8177***  -0.382***  0.8721 / 0.8670 | | 296.7077***  -4.2434**  0.0224  0.9185 / 0.9117 | | -1230.433  46.0599  -0.5648  0.0023  0.9259/ 0.9163 | | 929.2284  -70.4532  1.7508  -0.0178  0.0000  0.9337/ 0.9216 |
| Myanmar | Intercept  RE  RE^2^  RE3  RE4  R^2^ / R^2^ Adjusted | 90.8679***  -0.9738***  0.8129 / 0.8054 | | 123.1407*  -1.8786  0.0062  0.8145/ 0.7991 | | -1480.96**  65.8139**  -0.9366**  0.0043**  0.8598/ 0.8415 | | -8659.952  468.2474  -9.3376  0.0817  -0.0003  0.8686/ 0.8448 |
| Nepal | Intercept  RE  RE^2^  RE3  RE4  R^2^ / R^2^ Adjusted | 73.4979***  -0.7847  0.9277 / 0.9248 | | 103.7848  -1.5633  0.0046  0.9283/ -0.9223 | | -2825.778***  104.0195***  -1.2597***  0.0050***  0.9615/ 0.9452 | | -6845.225**  310.5228**  -5.2148*  0.0385***  -0.0001  0.9565/ 0.9486 |
| Niger | Intercept  RE  RE^2^  RE3  RE4  R^2^ / R^2^ Adjusted | 11.7903***  -0.1251***  0.7225 / 0.7114 | | -21.9500  0.6958***  -0.0050*  0.7454 / 0.7241 | | -840.8095***  30.8643***  -0.3746***  0.0015***  0.8090/ 0.7840 | | 1517.622  -83.5278  1.6994  -0.0151  0.0000  0.8181/ 0.7850 |
| Senegal | Intercept  RE  RE^2^  RE3  RE4  R^2^ / R^2^ Adjusted | 25.43761***  -0.4252**  0.4734 / 0.4523 | | 122.9885***  -4.8756  0.0515  0.5995/ 0.5661 | | 737.6528**  1.0009*  -0.0071*  0.6548/ 0.6098 | | -1603.227  168.1553  -6.3678  0.1045  -0.0006  0.6642/ 0.6032 |
| Sudan | Intercept  RE  RE^2^  RE3  RE4  R^2^ / R^2^ Adjusted | 59.6358***  -0.6760***  0.8815 / 0.8767 | | 56.6216  -0.5877***  -0.0006***  0.8815 / 0.8716 | | 136.6045  7.9831  -0.1262  0.0006  0.8836/ 0.8684 | | -3116.101  183.9119  -3.9926  0.0380  -0.0001  0.8890/ 0.8689 |
| Tanzania | Intercept  RE  RE^2^  RE3  RE4  R^2^ / R^2^ Adjusted | 99.1837***  -1.0339  0.9653 / 0.9640 | | 121.7229  -1.6256  0.0033  0.9654 / 0.9625 | | -2814.962  96.6203  -1.0899  0.0040  0.9669/ 0.9626 | | -3179.577  136.3122  -2.1533  0.0149  -0.0000  0.9674/ 0.9615 |
| Togo | Intercept  RE  RE^2^  RE3  RE4  R^2^ / R^2^ Adjusted | 7.3851*  -0.0732***  0.4535 / 0.4316 | | 10.6159  -0.1642*  0.0006  0.4547 / 0.4092 | | -73.6689  3.3120  -0.0469  0.0002  0.4585/ 0.3879 | | -965.5185  52.7138  -1.0704  0.0096  -0.0000  0.4750/ 0.3795 |
| Uganda | Intercept  RE  RE^2^  RE3  RE4  R^2^ / R^2^ Adjusted | 85.1027***  -0.8804***  0.9420 / 0.9396 | | 301.7656  -4.1588  0.0176  0.9428 / 0.9381 | | 6189.478***  -196.1783***  2.0819***  -0.0073***  0.9637/ 0.9590 | | 3439.679  -188.4291***  3.7386  -0.0320  0.0001  0.9645/ 0.9581 |
| Zambia | Intercept  RE  RE^2^  RE3  RE4  R^2^ / R^2^ Adjusted | 61.3862***  -0.6635***  0.8202/ 0.8130 | | 456.8571*  -9.6245***  0.0519***  0.8357/ 0.8220 | | 2311.251  -74.6875  0.8085  -0.0029  0.8388/ 0.8178 | | 594.9081  -110.8337  3.4542  -0.0389  0.0001  0.8484/ 0.8209 |

Note: ***, **, * represent 1%, 5% and 10% significance level, respectively.

Source: Authors’ calculations based on data from World Bank.
