# Supplementary material for: Renewable energy as a solution to climate change: Insights from a comprehensive study across nations
Source: PLoS One. 2024 Jun 20;19(6):e0299807. doi: 10.1371/journal.pone.0299807 (PMC11189203; doi:10.1371/journal.pone.0299807)
Supplement: S7 Appendix — (DOCX) [file pone.0299807.s007.docx]

# S7 Appendix: Countries with the Highest Significant Positive Coefficients

| **Country** | **Economic Development Category** |  | **Simple Regression** | **Multiple Regression (Order 2)** | **Multiple Regression (Quadratic)** | **Multiple Regression (Polynomial)** |
| --- | --- | --- | --- | --- | --- | --- |
| China | DingE | Intercept  RE  RE^2^  RE^3^  RE^4^  R^2^ / R^2^ Adjusted | 13449.12***  -341.6103***  0.9021 / 0.8982 | 15422.9***  -578.3475***  5.9148  0.9106 / 0.9032 | 10979.09***  364.8231  -53.5310*  1.1049*  0.9231 / 0.9131 | 2766.797  2806.673  -294.7265  10.6767  -0.1314  0.9290 / 0.9161 |
| India | DingE | Intercept  RE  RE^2^  RE^3^  RE^4^  R^2^ / R^2^ Adjusted | 4699.446***  -76.1397***  0.9006 / 0.8966 | 8419.267***  -262.2506***  2.2395***  0.9390 / 0.9340 | -1452.636  490.2263  -16.4380**  0.1510**  0.9501 / 0.9436 | -79924.69***  8699.888***  -332.3255***  5.4499***  -0.0327**  0.9656 / 0.9594 |
| Germany | DE | Intercept  RE  RE^2^  RE^3^  RE^4^  R^2^ / R^2^ Adjusted | 974.8175***  -14.0065***  0.8789 / 0.8741 | 924.4883***  0.6342  -0.7511***  0.9210 / 0.9144 | 1016.802***  -40.8302***  3.9046***  -0.1495***  0.9508 / 0.9444 | 1001.019***  -31.3468  2.1358  -0.02276  -0.0030  0.9511/ 0.9422 |
| Japan | DE | Intercept  RE  RE^2^  RE^3^  RE^4^  R^2^ / R^2^ Adjusted | 1426.0320***  -40.1688***  0.5013 / 0.4814 | 1026.2950***  115.2393  -14.2108*  0.5587 / 0.5220 | 93.6381  656.062  -115.6331  6.1520  0.5818/ 0.5273 | 7609.199  -5151.899  1526.349  -195.0375  9.0258  0.6141 / 0.5439 |
| United States | DE | Intercept  RE  RE^2^  RE^3^  RE^4^  R^2^ / R^2^ Adjusted | 6572.887***  -131.1149***  0.5946 / 0.5784 | 4309.0360***  528.9035***  -44.1824***  0.7416/ 0.7201 | -1256.223  2947.999**  -378.9743**  14.8302**  0.7897 / 0.7623 | -24477.2  16417.1**  -3215.973**  272.2552**  -8.5116*  0.8222 / 0.7899 |
| Australia | DE | Intercept  RE  RE^2^  RE^3^  RE^4^  R^2^ / R^2^ Adjusted | 368.2556***  1.4988  0.0020/-0.0379 | 1611.0030***  -306.7489***  18.8129***  0.2856 / 0.2260 | -783.0340  -818.8508  80.7130  -2.4698  0.2986/ 0.2071 | -54658.5  26599.85  -4771.706  376.5399  -11.0286  0.3561 / 0.2390 |
| Canada | DE | Intercept  RE  RE^2^  RE^3^  RE^4^  R^2^ / R^2^ Adjusted | 320.8862  10.8336  0.0298 / -0.0090 | -3754.584  381.9718  -8.4470  0.0333 / -0.0473 | 598592  -82081.98  3753.884  -57.2038  0.0546/ -0.0687 | 142888.8  0  -1788.449  109.0616  -1.8698  0.0546 / -0.0688 |
| France | DE | Intercept  RE  RE^2^  RE^3^  RE^4^  R^2^ / R^2^ Adjusted | 559.7212***  -16.4951***  0.8689 / 0.8637 | 551.8368***  -15.1102  -0.0585  0.8690 / 0.8580 | -360.7272  224.3484  -20.5986  0.5763  0.8811 / 0.8656 | 1339.809  -368.956  55.8876  -3.7421  0.0901  0.8819 / 0.8605 |
| Italy | DE | Intercept  RE  RE^2^  RE^3^  RE^4^  R^2^ / R^2^ Adjusted | 537.7056***  -10.4697***  0.8095 / 0.8019 | 412.5909***  17.9807***  -1.2618***  0.9266 / 0.9205 | 176.3227***  95.0584***  -8.4978***  0.2053***  0.9737 / 0.9703 | 4.6447  166.3127***  -18.9067***  0.8280**  -0.0130*  0.9771/ 0.9729 |
| Poland | DE | Intercept  RE  RE^2^  RE^3^  RE^4^  R^2^ / R^2^ Adjusted | 360.9289***  -3.4997**  0.2160 / 0.1847 | 513.0673***  -38.6434**  1.9037**  0.3388 / 0.2837 | 1481.631***  -375.6195***  39.6631***  -1.3701***  0.6399/ 0.5929 | 4415.087***  -1745.293***  273.776***  -18.7056***  0.4700**  0.7258 / 0.6760 |
| Spain | DE | Intercept  RE  RE^2^  RE^3^  RE^4^  R^2^ / R^2^ Adjusted | 384.8757***  -7.5459***  0.5440 / 0.5257 | 358.1763***  -2.8553  -0.1846  0.5453 / 0.5074 | 900.7255**  -142.6443  11.2374  -0.2979  0.5852 / 0.5311 | 818.6815  -113.7808  7.5662  -0.0980  -0.0039  0.5853 / 0.5099 |
| United Kingdom | DE | Intercept  RE  RE^2^  RE^3^  RE^4^  R^2^ / R^2^ Adjusted | 590.7326***  -21.3813***  0.9532 / 0.9514 | 599.2145***  -27.3342***  0.5237  0.9570/ 0.9535 | 585.4759***  -12.8303  -2.4694  0.1631  0.9608 / 0.9557 | 613.7188  -51.1348*  10.5796  -1.3972  0.0604  0.9641/ 0.9576 |
| Mexico | DingE | Intercept  RE  RE^2^  RE^3^  RE^4^  R^2^ / R^2^ Adjusted | 709.8339***  -25.9936***  0.5844 / 0.5677 | -619.1534*  222.9645***  -11.4317***  0.7607 / 0.7408 | -7400.504***  2169.097***  -195.5829***  5.7408***  0.8335 / 0.8117 | -63534.78***  23524.11***  -3216.94***  194.1586***  -4.370441***  0.9165/0.9014 |
| Saudi Arabia | DingE | Intercept  RE  RE^2^  RE^3^  RE^4^  R^2^ / R^2^ Adjusted | 333.5483***  9695.746*  0.1050 / 0.0692 | -176.3411  79038*  -1921752*  0.2124 / 0.1468 | -3950.481***  790330.3***  -0.0000***  -0.0000***  0.4925 / 0.4263 | -3072.499  577108.6  -2.40e+07  4.32e+07  8.42e+09  0.4936/0.4016 |
| South Africa | DingE | Intercept  RE  RE^2^  RE^3^  RE^4^  R^2^ / R^2^ Adjusted | 562.4024***  -10.8262***  0.6484 / 0.6343 | 390.2147***  16.3486  -1.0096  0.6859 / 0.6597 | -628.9526*  274.2466***  -22.0158***  0.5485***  0.7816 / 0.7532 | -1043.57  414.2771  -39.20423  1.458473  -.0175695  0.7823/0.7427 |
| Turkey | DingE | Intercept  RE  RE^2^  RE^3^  RE^4^  R^2^ / R^2^ Adjusted | 617.8356***  -20.5526***  0.8062 / 0.7984 | 936.6481***  -62.1393***  1.2844**  0.8410 / 0.8277 | 164.0523  94.9096  -9.0089  0.2172  0.8515 / 0.8321 | -773.3302  353.5865  -35.0848  1.3558  -0.0182  0.8521 / 0.8252 |
| Ukraine | EiT | Intercept  RE  RE^2^  RE3  RE4  R^2^ / R^2^ Adjusted | 348.3705***  -19.9322  0.7300 / 0.7192 | 355.216***  -25.2898  0.6967  0.7325/ -0.7102 | 303.2798***  38.2772  -19.1018  1.7036  0.7720/ 0.7422 | 302.0104***  40.2475  -20.0281  1.8690  -0.0099  0.7720/ 0.7305 |
| Iran | DingE | Intercept  RE  RE^2^  RE^3^  RE^4^  R^2^ / R^2^ Adjusted | 348.4317***  180.8861  0.0780 / 0.0412 | -150.1831  1314.209*  -606.5885*  0.1792 / 0.1108 | 902.412  -2555.799  3789.088  -1559.837  0.2101 / 0.1071 | 1363.496  -4914.444  8077.658  -4862.236  911.2498  0.2106 / 0.0671 |
| Indonesia | DingE | Intercept  RE  RE^2^  RE^3^  RE^4^  R^2^ / R^2^ Adjusted | 911.4116***  -13.7174***  0.9789 / 0.9780 | 833.0899***  -8.8082**  -0.0708  0.9804 / 0.9787 | 945.2077***  -19.5220  0.2531  -0.0031  0.9806 / 0.9781 | 2435.273**  -210.7437  9.0919  -0.1780  0.0013  0.9821 / 0.9789 |
| Brazil | DingE | Intercept  RE  RE^2^  RE^3^  RE^4^  R^2^ / R^2^ Adjusted | 453.0028  -0.9547  0.0005 / -0.0395 | 5761.212  -237.8547  2.6385  0.0176 / -0.0643 | 111387.9  -7286.316  159.2055  -1.1577  0.0345 / -0.0914 | 30516. 54  0  -86.6637  2.525  -0.02066  0.0360/ -0.0898 |

Note: ***, **, * represent 1%, 5% and 10% significance level, respectively.

Source: Authors’ calculations based on data from World Bank.
