# Supplementary material for: Renewable energy as a solution to climate change: Insights from a comprehensive study across nations
Source: PLoS One. 2024 Jun 20;19(6):e0299807. doi: 10.1371/journal.pone.0299807 (PMC11189203; doi:10.1371/journal.pone.0299807)
Supplement: Attachment — Submitted filename: Response to Reviewers.docx [file pone.0299807.s008.docx]

**Author Responses to Reviewer Comments**

Dear editor and reviewers,

We would like to express our profound appreciation to the editor for giving us the opportunity to revise and resubmit the paper. We are also grateful to the reviewers for the constructive comments and suggestions made on our manuscript which we found very helpful in revising and improving our paper. Given below is a detailed description of how we have addressed each comment in the revised version of the paper.

| **Journal Requirements** | |
| --- | --- |
| Please ensure that your manuscript meets PLOS ONE’s style requirements, including those for file naming. The PLOS ONE style templates can be found at <https://journals.plos.org/plosone/s/file?id=wjVg/PLOSOne_formatting_sample_main_body.pdf> and  <https://journals.plos.org/plosone/s/file?id=ba62/PLOSOne_formatting_sample_title_authors_affiliations.pdf> | Thank you for your valuable feedback. We have taken your comments seriously and have diligently formatted the manuscript and all supplementary files in strict accordance with PLOS ONE's style templates. This includes adhering to the specific file naming conventions and ensuring that every aspect of our submission meets the journal's requirements. We are fully committed to ensuring that our paper aligns with PLOS ONE's style guidelines. |
| We note that Figures 2(A), 2(B) and 3(A), 3(B) in your submission contain map images which may be copyrighted. All PLOS content is published under the Creative Commons Attribution License (CC BY 4.0), which means that the manuscript, images, and Supporting Information files will be freely available online, and any third party is permitted to access, download, copy, distribute, and use these materials in any way, even commercially, with proper attribution. For these reasons, we cannot publish previously copyrighted maps or satellite images created using proprietary data, such as Google software (Google Maps, Street View, and Earth). For more information, see our copyright guidelines: <http://journals.plos.org/plosone/s/licenses-and-copyright>.    We require you to either present written permission from the copyright holder to publish these figures specifically under the CC BY 4.0 license, or (2) remove the figures from your submission:    a. You may seek permission from the original copyright holder of Figures 2(A), 2(B) and 3(A), 3(B) to publish the content specifically under the CC BY 4.0 license.    We recommend that you contact the original copyright holder with the Content Permission Form (<http://journals.plos.org/plosone/s/file?id=7c09/content-permission-form.pdf>) and the following text:  I request permission for the open-access journal PLOS ONE to publish XXX under the Creative Commons Attribution License (CCAL) CC BY 4.0 (<http://creativecommons.org/licenses/by/4.0/>). Please be aware that this license allows unrestricted use and distribution, even commercially, by third parties. Please reply and provide explicit written permission to publish XXX under a CC BY license and complete the attached form.    Please upload the completed Content Permission Form or other proof of granted permissions as an "Other" file with your submission.    In the figure caption of the copyrighted figure, please include the following text: “Reprinted from [ref] under a CC BY license, with permission from [name of publisher], original copyright [original copyright year]."    b. If you are unable to obtain permission from the original copyright holder to publish these figures under the CC BY 4.0 license or if the copyright holder’s requirements are incompatible with the CC BY 4.0 license, please either i) remove the figure or ii) supply a replacement figure that complies with the CC BY 4.0 license. Please check copyright information on all replacement figures and update the figure caption with source information. If applicable, please specify in the figure caption text when a figure is similar but not identical to the original image and is therefore for illustrative purposes only.  The following resources for replacing copyrighted map figures may be helpful:    USGS National Map Viewer (public domain): <http://viewer.nationalmap.gov/viewer/>  The Gateway to Astronaut Photography of Earth (public domain): <http://eol.jsc.nasa.gov/sseop/clickmap/>  Maps at the CIA (public domain): <https://www.cia.gov/library/publications/the-world-factbook/index.html> and <https://www.cia.gov/library/publications/cia-maps-publications/index.html>  NASA Earth Observatory (public domain): <http://earthobservatory.nasa.gov/>  Landsat: <http://landsat.visibleearth.nasa.gov/>  USGS EROS (Earth Resources Observatory and Science (EROS) Center) (public domain): [http://eros.usgs.gov/#](http://eros.usgs.gov/)  Natural Earth (public domain): <http://www.naturalearthdata.com/> | Thank you for bringing to our attention the potential copyright issues with the article. In response, we have promptly removed the copyrighted choropleth map images (Figures 2(A), 2(B), 3(A), 3(B)) from our submission.  In place of these images, we have incorporated Tables 1 and 2, as well as Appendix S3 and Appendix S4 to thoroughly analyse the differences in averages between the 1995-2004 and 2012-2021 periods concerning renewable energy consumption (REC) and CO_2_ emissions. For a more in-depth interpretation of Table 1, you can find it in the manuscript between lines between 283 - 313.  “According to Table 1, the US has the highest CO_2_ emissions among the developing countries in both 1995-2004 and 2012-2021 periods. However, from 1995-2004 to 2012-2021, we can see a 10% reduction which has resulted in 5255.338 millions of tons. Even though developed economies generally tend to produce more CO_2,_ an interesting fact is that China and India can be identified as the countries with the highest and the 3^rd^ highest average CO_2_ emissions in the world in the 2012-2021 decade. This is due to large scale manufacturing initiatives they are carrying out [88]. Interestingly, Russia is also among the top 5 CO_2_ emitting countries despite being a transitional economy.  On the other hand, the least developed countries have significantly lower amounts of CO_2_ emissions, whereas the highest CO_2_ emitting country, Bangladesh, emits only 77.675 million tons. This is mainly because these countries lack the capacity for large-scale production thus, their energy usage is considerably low [89]. However even though the amount of emissions is significantly lower compared to the other economic development categories, Bangladesh emits 185% more CO_2_ emissions in 2012-2021 compared to the average from 1995-2004. All top 10 CO_2_ emitting countries in the least developed category emits at least 100% more CO_2_ in 2012-2021 decade in comparison to the average from 1995-2004.  When comparing the results from Table 1 and Appendix S3, it is evident that the top 10 CO_2_-emitting countries in each category are almost the same in both periods. However, the emissions have increased significantly in all the categories except for developed category and a few countries from transitional economies. This shows that the countries responsible for the high levels of CO_2_ have remained constant for the past 25 years, and the negative impact has been increasing over the years. The most significant difference can be observed in China. It averages 10290.07 million tons within 2012-2021 which is 165% more of China’s CO_2_ emissions from 1995-2004, the highest CO_2_ emissions in that period. The next most considerable increment can be observed in India, with 944.87 million tons on average from 1995-2004 to an average of 2365.84 million tons between 2012-2021 which is an 150% increment over the two decades. US, Russia and Japan are in the top 5 CO_2_-emitting countries, emitting 5255.338, 1664.01 and 1186.1 million tons, respectively. The increases suggest that these countries should focus on cleaner energy sources to reduce their excess carbon footprint to ensure that the effects of climate change and global warming would not be catastrophic in the long run.”  For a detailed analysis of Table 2, please refer to the manuscript between lines 317 - 343.  “According to Table 2 and Appendix S4 the REC as a percentage of TEC is constantly the highest in the least developed countries in both periods, compared to other economies. However, there was a clear decreasing trend in the REC as a percentage of TEC moved towards 2012-2021, except in the developed economies. Although developed nations consume more RE, the REC as a percentage of TEC is lower than the least developed economies as the energy consumption from non-renewable energy sources are higher. However, the usage levels of RE have increased with time, demonstrating a dedication to sustainable energy practices.  Malta, Macao, United Kingdom, Belgium, Netherlands, Ireland, Cyprus, and Japan were the countries with developed economies with the lowest REC as a percentage of TEC for both periods (Table 2, Appendix S4). However, a significant rise for 2012–2021 was visible, specifically in Malta, Macao, United Kingdom and Belgium where their percentage increment is 19813%, 1035%, 794% and 545%. For the developing economies, Algeria, Bahamas, Iraq, Iran, Hong Kong, Saudi Arabia, and Singapore are highlighted as countries with the lowest REC as a percentage of TEC for both periods (Appendix S4). Saudi Arabia consistently recorded the lowest value for the REC as a percentage of TEC in both periods. It is the country with the lowest average REC as a percentage of TEC in the world in 2012-2021 period although it showed a slight increase for the period of 2012–2021. For transitional economies, most of the countries show an increase in the REC as a percentage of TEC while Kazakhstan, Russia, Kyrgyzstan and Georgia show a 18%, 9%, 23& and 44% decrease respectively. Among the least developed economies, Mauritius shows the lowest average REC as a percentage of TEC between 2012-2021 period with a significant decrease from 26.18% to 8.89% from 1995-2012 to 2012-2021. Similar drops were observed in all 10 countries with the lowest average REC in least developed countries. However, developing and least-developed nations still lag in implementing RES. These tables demonstrate the necessity of expediting the efforts and international collaboration to encourage the use of RE worldwide, especially in areas where consumption is still significantly low. Promoting and investing in RE systems is essential to mitigate climate change and create a more sustainable future.” |
| **Editor Comments** | |
| Please revise your paper according to reviewers' reports and mark into color all changes you will make into the manuscript. Also prepare a response letter for reviewers. | Thank you for your valuable feedback. We have thoroughly reviewed the reviewers' reports and incorporated the necessary revisions into the manuscript. In response to your request, we have marked all changes in colour within the document. Additionally, we have prepared a response letter to address each of the reviewers' comments. These responses are included in this document for your convenience. |
| We note that one or more reviewers has recommended that you cite specific previously published works. As always, we recommend that you please review and evaluate the requested works to determine whether they are relevant and should be cited. It is not a requirement to cite these works. We appreciate your attention to this request. | Thank you for your comment. We have carefully reviewed the suggested articles and have incorporated the majority of them into the manuscript's references. These additions have been made because they are highly relevant to the focus area of our study. We appreciate your guidance in this regard and believe that these citations will enhance the quality and comprehensiveness of our work. |
| **Reviewer’s Comments** | |
| 1. Is the manuscript technically sound, and do the data support the conclusions?  The manuscript must describe a technically sound piece of scientific research with data that supports the conclusions. Experiments must have been conducted rigorously, with appropriate controls, replication, and sample sizes. The conclusions must be drawn appropriately based on the data presented.  Reviewer #1: No  Reviewer #2: Yes | Well noted with thanks. The comments have been integrated in the revised manuscript. |
| 2. Has the statistical analysis been performed appropriately and rigorously?    Reviewer #1: No  Reviewer #2: Yes | Thank you for your feedback. The response was well considered. |
| 3. Have the authors made all data underlying the findings in their manuscript fully available?  The PLOS Data policy requires authors to make all data underlying the findings described in their manuscript fully available without restriction, with rare exception (please refer to the Data Availability Statement in the manuscript PDF file). The data should be provided as part of the manuscript or its supporting information or deposited to a public repository. For example, in addition to summary statistics, the data points behind means, medians and variance measures should be available. If there are restrictions on publicly sharing data—e.g. participant privacy or use of data from a third party—those must be specified.    Reviewer #1: Yes  Reviewer #2: Yes | Duly noted with thanks. The authors have ensured that all relevant data files have been made available with the manuscript. |
| 4. Is the manuscript presented in an intelligible fashion and written in standard English?  PLOS ONE does not copyedit accepted manuscripts, so the language in submitted articles must be clear, correct, and unambiguous. Any typographical or grammatical errors should be corrected at revision, so please note any specific errors here.    Reviewer #1: Yes  Reviewer #2: Yes | Thank you for your positive comments. Necessary changes to the language and writing style have been incorporated into the manuscript. |
| **Reviewer #1** | |
| Thank you very much for inviting me to assess the above-mentioned manuscript submitted to Plos One. From the research topic, this paper attempts to examine "Renewable Energy as a Solution to Climate Change: Insights from a Comprehensive Study Across Nations" approaches. | Thank you very much for your assessment of the manuscript titled 'Renewable Energy as a Solution to Climate Change: Insights from a Comprehensive Study Across Nations.' We appreciate your thoughtful comments and the time you've dedicated to reviewing our work. The authors are truly grateful for the feedback, which will undoubtedly contribute to enhancing the quality of the manuscript. |
| I suggest authors rewrite the abstract to make it more constructive. Abstract should have at least one sentence per each: context and background, motivation, hypothesis, methods, results, conclusions. | Thank you for your valuable feedback. We have revised the abstract to better align with the suggested structure, ensuring a more comprehensive and constructive summary of our research.  In the updated abstract (lines 24 - 35), we have provided a clear breakdown as follows:  Context and Background: “Without fundamentally altering how humans generate and utilise energy, there is no effective strategy to safeguard the environment”  Motivation: “The motivation behind this study was to analyse the effectiveness of renewable energy in addressing climate change, as it is one of the most pressing global issues.”  Hypothesis: “This study involved the analysis of panel data covering 138 nations over a 27 year period, from 1995 to 2021, making it the latest addition to the existing literature.”  Methods: “We examined the extent of the impact of renewable energy on carbon dioxide over time using panel, linear, and non-linear regression approaches.”  Results: “The results of our analysis, revealed that the majority of countries with the exception of Canada, exhibited a downward trend, underscoring the potential of increasing renewable energy consumption as an effective method to reduce carbon dioxide emissions and combat climate change.”  Conclusions: "Furthermore, to reduce emissions and combat climate change, it is advisable for nations with the highest carbon dioxide emissions to adopt and successfully transition to renewable energy sources.”  We believe these revisions will provide a clearer and more structured overview of our research. Once again, we appreciate your valuable input. |
| Intro & Literature: i) The introduction part of the study needs improvement and story flow and the authors need to give proper contributions to their study. ii) There is a need to do a more rigorous and systematic literature review. Give the touch of more environmental theory See and kindly add following paper to the references <https://doi.org/10.1080/15567249.2016.1263251>; <https://doi.org/10.1007/s13132-011-0075-2>; <https://doi.org/10.1007/s12667-010-0018-1>; <https://doi.org/10.1007/s11356-019-04514-6>; <https://doi.org/10.1007/s11356-021-16720-2>; <https://doi.org/10.1007/s11356-021-12993-9>; <https://doi.org/10.1007/s11356-023-26675-1>; <https://doi.org/10.1007/s11356-023-28977-w>; <https://doi.org/10.1108/IJCHM-10-2022-1201>; <https://doi.org/10.1007/s11356-023-29020-8> | Thank you for your insightful feedback on the introduction and literature review of our manuscript. We have taken your suggestions seriously and made significant improvements to these sections.  We've enhanced the flow of the introduction to create a more cohesive and logical narrative. Additionally, we have clearly outlined the contributions of our study, which are now elaborated on in lines 89 – 112.  “This study contributes to the existing literature in several fronts. Firstly, it analyses each country separately and categorises them based on their level of economic development as developed, developing, least developed, and transitional economies. Despite extensive research on the relationship between CO_2_ emissions and RE of developed and developing economies, a significant gap in the literature exists regarding the context of least developed and transitional economies. Focusing on understudied areas, our research aims to contribute to this limited body of literature by addressing the context of least developed and transitional economies to provide valuable insights for policymakers. Secondly, panel regression, linear regression and non-linear regression techniques are used in this study, allowing for a more comprehensive analysis of both linear and non-linear relationships between CO_2_ emissions and RE and the level of the impact on the respective variables over a period. This is important in understanding how the relationship between CO_2_ emissions and RE has evolved across different countries and accurately identifying patterns and changes. Thirdly, this study visualises the top 12 CO_2_ emitting countries, and the relationship between REC and CO_2_ emissions in each country in a single graph allowing the users to identify the countries who negatively impacts the environment the most and how they can reduce those effects by transitioning to RES. Finally, this study represents the latest addition to the current literature, analysing panel data from 1995 to 2021 over 27 years covering 138 nations. While previous research has focused on individual countries or a limited number of countries, there need to be more comprehensive global studies to provide a thorough understanding of the worldwide relationship between CO_2_ emissions and RE for a significant period. Therefore, this study provides a broader analysis based on a more substantial number of countries considering a more comprehensive period using the latest available data to obtain a more accurate depiction of the global status of the relationship between two variables helping for more precise decision-making.”  We have strengthened our literature review by incorporating a substantial number of the suggested articles into our manuscript. These additions can be found in lines 50, 65, 157 and 174.  These additional references not only enrich the theoretical framework but also provide a more comprehensive understanding of the topic, reinforcing the scholarly depth of our study.  “Thus, the adverse environmental impacts, as well as the factors which influence CO_2_ emissions have remained an important subject of worldwide discussions among the academic community for many years [7, 8].”  “Hence, the academic community has conducted multiple studies on achieving sustainability goals, carbon neutrality, alternative options for fossil fuels such as natural gases, RE and their impact towards environmental sustainability [12-16].”  “Moreover, multiple studies based on Organization for Economic Cooperation and Development (OECD) countries concluded that REC and investment in RE reduces CO_2_ emissions while helping to achieve environmental sustainability [16, 33, 34].”  “Further, multiple studies about CO_2_ emissions and RE in the United States collectively concluded that despite the rising public acceptance of the fact that burning of fossil fuels is a major reason for climate change and the usage of RE can mitigate climate change, the levels of REC of the United States cannot significantly contribute to CO_2_ emissions reduction yet [38-43].” |
| Take out the footnotes from text. | Thank you for your feedback. Footnotes are only present on the title page to list the affiliations of each author, in accordance with the PLOS ONE journal guidelines. No other footnotes are used in the text. You can refer to the provided template for reference as below.  <https://storage.googleapis.com/plos-published-prod/3fac/PLOS%20Affiliations%20Formatting%20Guidelines.pdf?X-Goog-Algorithm=GOOG4-RSA-SHA256&X-Goog-Credential=wombat-sa%40plos-prod.iam.gserviceaccount.com%2F20231013%2Fauto%2Fstorage%2Fgoog4_request&X-Goog-Date=20231013T055223Z&X-Goog-Expires=86400&X-Goog-SignedHeaders=host&X-Goog-Signature=705d17d35a38e1d4f8d1b2e81bd70814fa0dcc65a5344eeea1216daec8dda71638551d45cc8692306840241f7d5f7bff45a1026009a89107336696d54b0610ee34ab059be3131cd9b8ab70a6eb9ffe65fea0eda81eab5f1f33ca752ec79779eb2c526e0bccfa5440ff62104cf2ad3d06066a88536a6b2d6de2d8835435ea9e6d91ed3fd6764af2c08a785d400b729ae06ef7e8f2e62273ca8fe5d13e0d8e19118e6dbda2eb4d29b902ef9c7fee211f58c24b1f661fc7ad2089d3d7a285973c8602290ca129f1536386cefcdc47313969c1e8bf17ba10452a590b8a9c7d6458b84887955627e3068f216685ea93ecf8edf14972a7df5a8e544966a5d9ff0db7e6> |
| Policy recs. are a bit vague and not convincing to the reader. | Thank you for your valuable input, and we appreciate your attention to the policy recommendations. We have taken your feedback seriously and have made significant improvements to the policy recommendations section.  The policy recommendations have been updated to provide more specific and convincing guidance for each country, considering all 12 of the highest CO_2_-emitting nations. These revisions aim to offer a more robust and actionable set of recommendations that address the challenges and opportunities presented by our study.  We believe that these enhancements have made our policy recommendations more concrete and persuasive, and we are grateful for your insights in this regard and these changes can be found between lines 467 -537.  “Transitioning towards RE is a complex exercise. It requires comprehensive policy measures covering economic, social, and technological factors which is more challenging, particularly for countries heavily dependent on fossil fuels for economic activities and energy security. Further, a collective effort of the nations to reduce CO_2_ emissions would significantly impact global climate mitigation. This study suggests the following recommendations for countries with the highest CO_2_ emissions to embrace a successful transition towards RES, which helps to mitigate CO_2_ emissions and combat climate change.  Developed countries with a strong economic background such as Germany, Italy, Japan, United Kingdom, and Unites States must invest in research and development initiatives focusing on advanced RET and their integration into the energy grid, which will help overcome technological barriers and reduce the initial costs associated with RE implementation. The research and development initiatives of the developed countries with less REC as a percentage of TEC such as Germany, Japan, United Kingdom, and United States must focus on developing and modernising energy infrastructure to accommodate a higher share of RES. These expansions must accommodate smart grid technology implementation and energy storage solutions to ensure a resilient and reliable energy supply. However, developed economies which shows a positive relationship between REC and CO_2_ emissions such as Canada, must invest in alternative backup systems for intermittent renewable sources. Such efforts can prevent increased CO_2_ emissions and must maintain a balanced energy mix integrating RE and fossil fuel sources to ensure CO_2_ emission reductions are maintained. Furthermore, these developed countries can consider RE exports to neighbouring countries which will strengthen the economic relationships and support to reduce emissions. Also, governments must invest in research and development of RET enhanced energy production and improved energy storage, distribution, and grid management which facilitates energy storage systems to store excess energy during peak production and release it during high demand. Electrochemical energy storage technologies are one of the most suitable applications for RE due to their higher efficiency and flexible designs. However, RE storage faces challenges in scalability and cost-effectiveness; thus, it is ideal for developed economies. Also, floating solar panels on reservoirs and offshore wind farms can be recommended for countries with limited land resources such as Italy and Japan [91]. Moreover, governments must highly encourage the adoption of electric vehicles and support the rapid development of electric vehicle charging infrastructure to reduce CO_2_ emissions from the transportation sector. Furthermore, developed economies can consider satellite observations of CO_2_ emissions to get more accurate and timely data.  For the developing countries such as China, India, Indonesia, Mexico, South Africa, and Turkey, where increased REC has caused a substantial reduction in CO_2_ emissions, the investments and resource allocation towards RE projects should be increased. They can also gradually minimize the investments towards fossil fuel industries to accelerate RES adoption to be economically competitive. This can enhance energy security by improving energy independence and reducing the risk of being vulnerable to price fluctuations in global fossil fuel markets. Investing in advanced RES innovation will help reduce the costs associated with RE deployment and improve the efficiency and scalability of RES. Further, advanced Carbon Capture, Utilization and Storage technologies should be implemented on larger scales among countries such as China and India. Countries with rich ecosystems such as China, India, Mexico and Indonesia can enforce policies promoting afforestation and reforestation as the terrestrial and marine ecosystems act as CO_2_ reservoirs which could increase carbon sequestration and mitigate climate change. Moreover, developing economies with modest REC as a percentage of TEC such as Mexico and Turkey must further diversify the energy mix by including more RES, to reduce dependence on fossil fuels. The governments and local industrial experts of above developing countries must form international collaborations and partnerships with governments with developed economies, and foreign industrial experts to share best practices, technological knowledge, and financial resources for RE development.  Moreover, all the governments of the above mentioned 12 highest CO_2_ emitting countries must establish clear objectives and timelines for the implementation of RES. These objectives and timelines must be developed as a long-term energy transition strategy to avoid the usage of fossil fuels and maximise REC. These strategies should be periodically reviewed and methodically adjusted based on evolving technological, economic, and environmental considerations. Finally, supportive policy frameworks that facilitate the transition to RE should be implemented. Moreover, these governments must endeavour to attract private and public investment by providing clear regulatory frameworks, financial incentives for RE projects, and launching public awareness campaigns to educate citizens about the benefits of RE and the importance of carbon emission reduction. Building public support can drive demand for RES and encourage more outstanding political commitment. Moreover, implementation of carbon pricing mechanisms such as carbon taxes or to provide economic incentives for reducing CO_2_ emissions must be enabled. Also, governments must invest in re-training programs to transition employees involved in fossil fuel industries to RE sectors to build a capable workforce that can support the RE transition and be skilled in designing, installing, operating, and maintaining RE systems.  Finally, all countries must make significant and consistent efforts to mitigate CO_2_ emissions, combat climate change, and foster sustainable economic growth to ensure a brighter future by diligently implementing strategies and embracing comprehensive approaches to transit into using RES for energy production. |
| It would be appropriate to indicate future research directions and limitations of this at the end of the conclusion section just before references. Need clear future recommendation/implementation in the context of uncertainty <https://doi.org/10.1007/s00477-023-02452-x>; <https://doi.org/10.1177/1354816619888346> | Thank you for your valuable feedback. We have taken your suggestion to heart and have included a section on limitations and future research directions, just before the references.  **Limitations of the study:** One of the primary limitations of this research was the unavailability of data on various renewable energy sources during the study period. This limitation constrains our ability to explore the specific impacts of solar power, bioenergy, and geothermal power on economic growth. Future studies in this area should aim to overcome this data limitation to provide a more comprehensive analysis.  **Future research directions:** In the context of uncertainty, future research should delve into the relationship between renewable energy sources, particularly wind and solar energy, and their effects on additional greenhouse gases and ecological footprints, in addition to CO_2_ emissions. This expanded focus will contribute to a more holistic understanding of the environmental impact of renewable energy adoption.  We believe that by addressing these limitations and pursuing these future research avenues, we can further advance our knowledge and understanding of the complex interplay between renewable energy and environmental sustainability. The changes can be found between lines 539 and 544.  “As this paper focused on identifying the relationship from RE to CO_2_ emissions over 27 years for 138 nations, the primary limitation was the absence of the source of RE during the study period. Therefore, it would be challenging to explore more in future studies about solar power, bioenergy, and geothermal power affect economic growth. Moreover, future studies should evaluate the connection between RE, particularly wind and solar energy, and additional GHG and ecological footprint in addition to CO_2_ emissions.” |
| **Reviewer #2** | |
| 1)Major findings or trends found as a result of the study.  - A summary of your interpretations and conclusions. | Thank you for your suggestion. We have addressed this by summarizing the major findings, interpretations, and conclusions.  In this section, we highlight the significance of renewable energy and its potential to address pressing issues such as the energy crisis and rising CO_2_ emissions. Our study distinguishes itself by offering a comprehensive analysis that spans a wide range of countries over an extended period, utilizing the most current data available, and addressing crucial research gaps.  The findings of our study demonstrate a clear trend where nearly all countries, with the exception of Canada, exhibit a decline in CO_2_ emissions. This points to the promising role of renewable energy in reducing global emissions and the importance of increased investment in renewable sources for economic and environmental benefits.  Our conclusions underscore the positive relationship between renewable energy and CO_2_ emissions in Canada, emphasizing the long-term advantages of renewable energy investment. We also highlight the pivotal role of policy promotion in fostering renewable energy growth, ultimately leading to sustainable development that balances economic growth with environmental considerations.  In summary, our study showcases the potential of renewable energy as a solution to pressing global challenges, and we appreciate your input in helping us clarify this in the manuscript between lines 449 – 465.  “The significance of RE has been emphasised through this study, considering the energy crisis and the risks of continually increasing CO_2_ emissions on the environment. Previous studies have concentrated on a single country or a few nations. In contrast, this study offers a more comprehensive analysis based on a substantial number of countries, covering a more longitudinal time range, utilising the most recent data and addressing the research gaps. Based on the results, only 12 nations with the most CO_2_ emissions were chosen to study the relationship between CO_2_ and RE due to the adverse environmental impacts.  The study’s findings, which showed that almost all nations except Canada had a declining trend, highlighted the possibility that an increment in RES might reduce the global effect of emissions. Given the status of many nations, increasing their investment in RES would be highly advantageous on both an economic and environmental context as such sources restrict the emissions of GHG. Further, the current analysis provides evidence that Canada shows a positive relationship between the variables. Long-term reduction of CO_2_ emissions is a benefit of RE. The significance that policy promotion plays in the growth of RE should be taken seriously by the government. Only through analysing RE policies, one of the clean energy resources, is it feasible to figure out sustainable development, which results from the consideration of the environment when guaranteeing economic growth.” |
| All abbreviations must be introduced at first mention. Consider the abstract independently. Only recheck it, please. | Thank you for your valuable feedback. We have duly considered your suggestion regarding the introduction of abbreviations and made the necessary updates. Specifically, we have ensured that all abbreviations are introduced at their first mention in the main text (excluding the abstract).  In response to your comment, we have introduced two new abbreviations: 'Renewable Energy Production (REP)' and 'Renewable Energy Technologies (RET)' to enhance clarity and understanding for the readers.  We appreciate your attention to detail, and we believe these updates enhance the readability of the manuscript. |
| The Introduction should have valuable aspects such as motivation, problem, challenges, the significance of the study…etc. All these are disappearing. Therefore, the introduction needs to clarify the (1) motivation, (2) challenges, (3) contribution, (4) objectives, and (5) significance/implication of this systematic. All the information (should be) presented in sequence idea. | Thank you for your valuable feedback, which we highly appreciate. In response to your suggestions, we have made several important updates to the Introduction section to enhance its structure and clarity. Here's a breakdown of the changes made:  Motivation (Lines 63-67): We have now explicitly articulated the motivation for the study. This motivation is explained in the lines as follows:  “These concerns ignited the motivation to conduct this study to identify the effectiveness of Renewable Energy (RE) usage on reducing the dependency on fossil fuels which allows a significant decrease in CO2 emissions and therefore mitigating the impacts it has on the environment.”  Challenges (Lines 59-61): We have introduced a section that highlights the challenges the world is currently facing due to the focused areas of our study. The added lines now read:  “If the usage of fossil continues resulting further increase of CO_2_ emissions [10] it can cause substantial harm to the environment and the living organisms. Therefore, switching from traditional energy to RE is of utmost importance.”  Contribution and Significance (Lines 89-112): We have included a detailed explanation of the study's contribution and significance in these lines. This now reads as follows:  “This study contributes to the existing literature in several fronts. Firstly, it analyses each country separately and categorises them based on their level of economic development as developed, developing, least developed, and transitional economies. Despite extensive research on the relationship between CO_2_ emissions and RE of developed and developing economies, a significant gap in the literature exists regarding the context of least developed and transitional economies. Focusing on understudied areas, our research aims to contribute to this limited body of literature by addressing the context of least developed and transitional economies to provide valuable insights for policymakers. Secondly, panel regression, linear regression and non-linear regression techniques are used in this study, allowing for a more comprehensive analysis of both linear and non-linear relationships between CO_2_ emissions and RE and the level of the impact on the respective variables over a period. This is important in understanding how the relationship between CO_2_ emissions and RE has evolved across different countries and accurately identifying patterns and changes. Thirdly, this study visualises the top 12 CO_2_ emitting countries, and the relationship between REC and CO_2_ emissions in each country in a single graph allowing the users to identify the countries who negatively impacts the environment the most and how they can reduce those effects by transitioning to RES. Finally, this study represents the latest addition to the current literature, analysing panel data from 1995 to 2021 over 27 years covering 138 nations. While previous research has focused on individual countries or a limited number of countries, there need to be more comprehensive global studies to provide a thorough understanding of the worldwide relationship between CO_2_ emissions and RE for a significant period. Therefore, this study provides a broader analysis based on a more substantial number of countries considering a more comprehensive period using the latest available data to obtain a more accurate depiction of the global status of the relationship between two variables helping for more precise decision-making.”  Objectives (Lines 113-121): We have added a specific statement that outlines the primary objectives of the study, which now reads:  “The study’s primary objective is to investigate the relationship between RE and CO_2_ emissions in various countries to make the said four contributions to the existing literature while identifying the countries who negatively impact the environment due to their high CO_2_ emissions and how they can reduce such impacts by transitioning towards RES. As the interrelationship and overall social impact between CO_2_ emissions and RE remain to be fully understood by the industry professionals, policymakers, and academic community despite the substantial amount of research, this study will provide accurate and reliable insights to make informed decisions regarding the promotion of RE and mitigate CO_2_ emissions tailored to each country’s unique context.”  We believe these enhancements provide a more structured and informative Introduction section that effectively addresses the motivation, challenges, contribution, objectives, and significance of the study, as suggested. Thank you for your valuable input, which has been invaluable in improving the clarity and coherence of our manuscript. |
| I can see that the presented tables lack analysis. The authors should add a brief description and provide an analysis of the results in these tables to be clear for the readers. | Thank you for your valuable feedback, which has been instrumental in enhancing the clarity and comprehensibility of our work. We acknowledge the importance of providing a detailed analysis of the results presented in the tables to benefit our readers. In response to your suggestion, we have added a more comprehensive explanation of the results in Table 3.  In Table 3, the results from various statistical tests are presented to guide the selection of the appropriate econometric model. The analysis of these results is essential in determining the optimal model for our dataset. Here is a breakdown of the findings from the different tests:  **F Test:** The results indicate that the null hypothesis (H_0_) of all five economic development categories has been rejected. This rejection suggests that the Fixed Effects (FE) model is more suitable for our analysis.  **Hausman Test:** The results of the Hausman test further guide the model selection. The FE model is recommended for the developing economies, while for the least developed economies, the results are significant at the 10% level, indicating that the FE model is also suitable in this case.  **Breusch Pagan LM Test:** The outcome of the Breusch Pagan LM test suggests that the Random Effects (RE) model should be employed for all five economic development categories.  These changes now read as follows (Lines 349-356):  “According to the results from the F test, H_0_ of all five economic development categories were rejected indicating that the FE model is more suitable. Moving on to the Hausman test, the FE model is suggested for the developing and least developed economies as the results are significant at the 1% and 10% levels, respectively. The outcome of the Breusch Pagan LM test state that the RE model should be employed for all five categories. Considering the findings, the FE model was utilised for the developing and least developed economies whilst the RE model was employed for all countries, developed and economies in transition.”  We believe that this enhanced analysis of the results in Table 3 provides a clearer understanding of our methodological choices and ensures that our readers can better comprehend the rationale behind our selection of econometric models. Thank you once again for your valuable input. |
| What are the limitations of the presented study? I missed this part. | Thank you for your feedback. We have taken note of your suggestion and expanded on the limitations, as detailed in lines 539 - 544.  “As this paper focused on identifying the relationship from RE to CO_2_ emissions over 27 years for 138 nations, the primary limitation was the absence of the source of RE during the study period. Therefore, it would be challenging to explore more in future studies about solar power, bioenergy, and geothermal power affect economic growth. Moreover, future studies should evaluate the connection between RE, particularly wind and solar energy, and additional GHG and ecological footprint in addition to CO_2_ emissions.” |
| Double-check for grammar, typos, and abbreviations. | Thank you for your comment. The manuscript has been carefully reviewed, and we have made the necessary corrections for spelling mistakes, grammar, and abbreviations. |
| Improve the English language by profession. Improve your style in academic writing. | Thank you for your feedback. We have taken your comments into account and made significant improvements to the professionalism of the language used in the manuscript. |
| A lot of studies have been published in the literature. What is new in this work? The authors should prove the novelty and originality of the presented work. | Duly noted! The novelty and originality of the study are presented in lines 89 - 112, where we outline several unique contributions to the existing literature. This research not only fills critical gaps by analysing the context of least developed and transitional economies but also employs a variety of regression techniques for a more comprehensive analysis, offers visualizations to enhance understanding, and contributes to the existing literature by providing a broader global perspective. These aspects collectively make our study a valuable addition to the field, offering precise insights for policymakers and researchers.  “This study contributes to the existing literature in several fronts. Firstly, it analyses each country separately and categorises them based on their level of economic development as developed, developing, least developed, and transitional economies. Despite extensive research on the relationship between CO_2_ emissions and RE of developed and developing economies, a significant gap in the literature exists regarding the context of least developed and transitional economies. Focusing on understudied areas, our research aims to contribute to this limited body of literature by addressing the context of least developed and transitional economies to provide valuable insights for policymakers. Secondly, panel regression, linear regression and non-linear regression techniques are used in this study, allowing for a more comprehensive analysis of both linear and non-linear relationships between CO_2_ emissions and RE and the level of the impact on the respective variables over a period. This is important in understanding how the relationship between CO_2_ emissions and RE has evolved across different countries and accurately identifying patterns and changes. Thirdly, this study visualises the top 12 CO_2_ emitting countries, and the relationship between REC and CO_2_ emissions in each country in a single graph allowing the users to identify the countries who negatively impacts the environment the most and how they can reduce those effects by transitioning to RES. Finally, this study represents the latest addition to the current literature, analysing panel data from 1995 to 2021 over 27 years covering 138 nations. While previous research has focused on individual countries or a limited number of countries, there need to be more comprehensive global studies to provide a thorough understanding of the worldwide relationship between CO_2_ emissions and RE for a significant period. Therefore, this study provides a broader analysis based on a more substantial number of countries considering a more comprehensive period using the latest available data to obtain a more accurate depiction of the global status of the relationship between two variables helping for more precise decision-making.” |
| Rewrite the Conclusion and consider the following comments:  - Highlight your analysis and reflect only the essential points for the paper.  - Mention the benefits  - Mention the implication in the last of this section. | Thank you for your feedback. The comments have been integrated into the conclusion section (lines 448 - 465).  “The significance of RE has been emphasised through this study, considering the energy crisis and the risks of continually increasing CO_2_ emissions on the environment. Previous studies have concentrated on a single country or a few nations. In contrast, this study offers a more comprehensive analysis based on a substantial number of countries, covering a more longitudinal time range, utilising the most recent data and addressing the research gaps. Based on the results, only 12 nations with the most CO_2_ emissions were chosen to study the relationship between CO_2_ and RE due to the adverse environmental impacts.  The study’s findings, which showed that almost all nations except Canada had a declining trend, highlighted the possibility that an increment in RES might reduce the global effect of emissions. Given the status of many nations, increasing their investment in RES would be highly advantageous on both an economic and environmental context as such sources restrict the emissions of GHG. Further, the current analysis provides evidence that Canada shows a positive relationship between the variables. Long-term reduction of CO_2_ emissions is a benefit of RE. The significance that policy promotion plays in the growth of RE should be taken seriously by the government. Only through analysing RE policies, one of the clean energy resources, is it feasible to figure out sustainable development, which results from the consideration of the environment when guaranteeing economic growth.” |
| Remove the duplication between abstract and Conclusion. | Thank you for your suggestion. The duplication between the abstract and conclusion has been resolved. The abstract (lines 24-35) now provides a concise overview of the study, while the conclusion (lines 448 - 465) offers a detailed summary of the findings and their implications.  Abstract - “Without fundamentally altering how humans generate and utilise energy, there is no effective strategy to safeguard the environment. The motivation behind this study was to analyse the effectiveness of renewable energy in addressing climate change, as it is one of the most pressing global issues. This study involved the analysis of panel data covering 138 nations over a 27 year period, from 1995 to 2021, making it the latest addition to the existing literature. We examined the extent of the impact of renewable energy on carbon dioxide over time using panel, linear, and non-linear regression approaches. The results of our analysis, revealed that the majority of countries with the exception of Canada, exhibited a downward trend, underscoring the potential of increasing renewable energy consumption as an effective method to reduce carbon dioxide emissions and combat climate change. Furthermore, to reduce emissions and combat climate change, it is advisable for nations with the highest carbon dioxide emissions to adopt and successfully transition to renewable energy sources.”  Conclusion - “The significance of RE has been emphasised through this study, considering the energy crisis and the risks of continually increasing CO_2_ emissions on the environment. Previous studies have concentrated on a single country or a few nations. In contrast, this study offers a more comprehensive analysis based on a substantial number of countries, covering a more longitudinal time range, utilising the most recent data and addressing the research gaps. Based on the results, only 12 nations with the most CO_2_ emissions were chosen to study the relationship between CO_2_ and RE due to the adverse environmental impacts.  The study’s findings, which showed that almost all nations except Canada had a declining trend, highlighted the possibility that an increment in RES might reduce the global effect of emissions. Given the status of many nations, increasing their investment in RES would be highly advantageous on both an economic and environmental context as such sources restrict the emissions of GHG. Further, the current analysis provides evidence that Canada shows a positive relationship between the variables. Long-term reduction of CO_2_ emissions is a benefit of RE. The significance that policy promotion plays in the growth of RE should be taken seriously by the government. Only through analysing RE policies, one of the clean energy resources, is it feasible to figure out sustainable development, which results from the consideration of the environment when guaranteeing economic growth.” |
