# Supplementary material for: Renewable energy as a solution to climate change: Insights from a comprehensive study across nations
Source: PLoS One. 2024 Jun 20;19(6):e0299807. doi: 10.1371/journal.pone.0299807 (PMC11189203; doi:10.1371/journal.pone.0299807)
Supplement: Attachment — Submitted filename: Response to Reviewers.docx [file pone.0299807.s009.docx]

**Authors Response to Editor and Reviewers Comments**

Dear editor and reviewers,

We appreciate the feedback provided on our manuscript to improve the overall quality of our work. Necessary changes have been made to the manuscript taking into account the comments provided. Your input has been incredibly helpful in revising and improving the quality of our work.

Please note that the line numbers referred to in this document are aligned with the revised manuscript which includes track changes.

Thank you once again for your valuable feedback.

| **Journal Requirements** | |
| --- | --- |
| Please review your reference list to ensure that it is complete and correct. If you have cited papers that have been retracted, please include the rationale for doing so in the manuscript text, or remove these references and replace them with relevant current references. Any changes to the reference list should be mentioned in the rebuttal letter that accompanies your revised manuscript. If you need to cite a retracted article, indicate the article’s retracted status in the References list and also include a citation and full reference for the retraction notice. | Thank you for your thoughtful review and constructive comments. We appreciate your diligence in ensuring the accuracy and completeness of our reference list.  Upon your suggestion, we meticulously reviewed our reference list to confirm its completeness and accuracy. We confirm that no retracted papers have been cited in the manuscript. In response to your guidance, we have made additional improvements to the reference list by incorporating eight recent references. The revised list now comprises a total of 98 references, up from the initial count of 90.  We would like to emphasize that every effort has been made to adhere to your recommendations, and we believe the reference list now aligns with the highest standards of scholarly accuracy.  Should you have any further concerns or require additional clarification, please do not hesitate to let us know. We look forward to your feedback on the revised manuscript.  Thank you for your time and valuable insights. |
| **Editor Comments** | |
| One or more of the reviewers has recommended that you cite specific previously published works. Members of the editorial team have determined that the works referenced are not directly related to the submitted manuscript. As such, please note that it is not necessary or expected to cite the works requested by the reviewer. | Thank you for providing us with the feedback regarding the recommendations from the reviewers. We appreciate the editorial team's clarification on the relevance of certain previously suggested citations.  Upon careful consideration, we have reviewed the 11 articles recommended by the reviewer. After a thorough assessment, we have identified that 8 of these articles align closely with the scope of our manuscript and contribute substantively to our study. Therefore, we have incorporated these relevant works into our revised manuscript.  We believe that these additions enhance the overall quality and depth of our work without deviating from the focus of our research. We trust that this decision aligns with the expectations of both the reviewers and the editorial team.  If you have any further concerns or require additional information, please feel free to let us know. We are committed to ensuring that our manuscript meets the highest standards of scholarly rigor. |
| **Reviewer Comments** | |
| 1. If the authors have adequately addressed your comments raised in a previous round of review and you feel that this manuscript is now acceptable for publication, you may indicate that here to bypass the “Comments to the Author” section, enter your conflict of interest statement in the “Confidential to Editor” section, and submit your "Accept" recommendation.  Reviewer #1: All comments have been addressed  Reviewer #3: (No Response) | Duly noted with thanks. |
| 2. Is the manuscript technically sound, and do the data support the conclusions?  The manuscript must describe a technically sound piece of scientific research with data that supports the conclusions. Experiments must have been conducted rigorously, with appropriate controls, replication, and sample sizes. The conclusions must be drawn appropriately based on the data presented.  Reviewer #1: Yes  Reviewer #3: Partly | Thank you for your feedback. The necessary changes have been incorporated into the manuscript. |
| 3. Has the statistical analysis been performed appropriately and rigorously?  Reviewer #1: Yes  Reviewer #3: Yes | Well noted with many thanks. |
| 4. Have the authors made all data underlying the findings in their manuscript fully available?  The PLOS Data policy requires authors to make all data underlying the findings described in their manuscript fully available without restriction, with rare exception (please refer to the Data Availability Statement in the manuscript PDF file). The data should be provided as part of the manuscript or its supporting information or deposited to a public repository. For example, in addition to summary statistics, the data points behind means, medians and variance measures should be available. If there are restrictions on publicly sharing data—e.g. participant privacy or use of data from a third party—those must be specified.  Reviewer #1: Yes  Reviewer #3: Yes | Well received. The data files have been made available as supplementary files. |
| 5. Is the manuscript presented in an intelligible fashion and written in standard English?  PLOS ONE does not copyedit accepted manuscripts, so the language in submitted articles must be clear, correct, and unambiguous. Any typographical or grammatical errors should be corrected at revision, so please note any specific errors here.  Reviewer #1: Yes  Reviewer #3: Yes | Thank you for your feedback in improving the quality of our work! |
| **Reviewer #1** | |
| Thank you for giving me the opportunity to read your paper. The paper “PONE-D-23-27314R1” is interesting for journal readers. My opinion is "accept" | We sincerely appreciate your time and effort in reviewing our manuscript, and we are thrilled to learn that you find our work interesting for journal readers. Your positive feedback is invaluable to us.  We have duly noted your recommendation for acceptance. If there are any specific revisions or suggestions you would like us to address before the final submission, please do not hesitate to let us know. We are committed to ensuring that our paper meets the highest standards and aligns with the expectations of the journal.  Once again, thank you for your thoughtful review and support. |
| **Reviewer #3** | |
| Renewable energy serves as a viable way to mitigate climate change. To substantiate this claim, it is necessary to include further information in the last paragraph describing how this research will make a distinctive contribution. | Thank you for your insightful comment. We have carefully incorporated the requested information into the manuscript, specifically in lines 347–351, 470–473, and 482–486.  In lines 347-351, we have added the following text to the last paragraph:  “Promoting and investing in RE systems is essential to mitigate climate change and create a more sustainable future. Increased utilisation of RES would significantly reduce pollution, environmental impact, and emissions contributing to the overall mitigation of climate change [21, 62].”  Additionally, in lines 470–473 and 482–486, the following statements have been included:  “Investing in initiatives to enhance the usage of RES would lead to a substantial reduction in non-renewable energy consumption, resulting in a significant decrease in CO_2_ emissions over the long term.”  “Furthermore, these findings also hold the potential to educate the general public on the pivotal role of RES in reducing CO2 emissions. Increased awareness in these areas is expected to drive positive changes in consumer behaviour and inspire innovative initiatives to address the effects of climate change.”  We believe that these additions strengthen the distinctive contribution of our research to the field. |
| - The current literature on renewable energy consumption and climate change lacks more recent information. The authors could additionally focus on environmental deterioration and carbon emissions. Here, I am providing some recommended research to enhance this section:  (1)-https://doi.org/10.1016/j.gr.2023.11.002 (2)-https://doi.org/10.1016/j.scitotenv.2023.168027 (3)-https://doi.org/10.1016/j.gr.2023.08.019 (4)- https://doi.org/10.1177/21582440211060829 (5)- https://doi.org/10.3390/su15129413 (6)-https://doi.org/10.3390/su15075916 (7)-https://doi.org/10.1007/s11356-023-25316-x (8)-https://doi.org/10.3390/en15197180 (9)-https://doi.org/10.1007/s11356-022-22775-6 (10)-https://doi.org/10.1007/s11356-022-19317-5 (11)- https://doi.org/10.1007/s11356-021-15481-2 | Thank you for providing valuable suggestions to enhance the literature on renewable energy consumption and climate change in our manuscript. We appreciate your effort in identifying relevant research articles. The following changes have been made to our manuscript in response to your recommendations:  Eight out of the eleven mentioned articles have been added to our literature, as they align closely with the variables addressed in our study. Our initial reference count of 90 has now increased to 98. The specific changes can be found in the manuscript at the following locations: lines 70-72, 73-75,168-171, 189-193, 200-204, and 347-351.  For instance  “Hence, minimising the usage of fossil fuel and optimising the use of RE would be the optimal option to attain carbon neutrality and sustainably fulfil the energy needs of humans as it can reduce CO2 emissions [18-20].”  “Transitioning to RE for energy production is essential as it helps reduce carbon emissions, mitigate climate change, enhance environmental sustainability, and foster long-term socio-economic benefits such as sustainable economic growth. [21-23]”  “Studies based on developed economies namely Canada, France, Japan, Netherlands, Spain, Sweden, Switzerland, United Kingdom, and the United States have concluded that RE reduces CO2 emissions and the use of RE is beneficial for environmental protection [41, 42].”  “Despite being recognised as the world’s largest CO2 emitter, China must focus on reducing its CO2 emissions by utilising RE. Multiple studies have concluded that the adoption of RE has a significant negative impact on CO2 emissions in China, contributing to the mitigating climate change [59-62].”  “Similar studies in China, Pakistan, Malaysia, and Indonesia have revealed that increasing and actively funding the proportion of power generated from RES is crucial to support sustainable development, climate change mitigation, and environmental protection [36, 62, 68, 69].”  “Promoting and investing in RE systems is essential to mitigate climate change and create a more sustainable future. Increased utilisation of RES would significantly reduce pollution, environmental impact, and emissions contributing to the overall mitigation of climate change [21, 62].”  We believe these additions strengthen the foundation of our study and contribute significantly to the existing literature. Once again, thank you for your insightful feedback and guidance. |
| Figure quality has to be improved further. | Thank you for your feedback. We acknowledge your comment regarding the figure quality. In response, we have made improvements to the figure by enhancing the colours and adjusting the font size to enhance legibility.  The revised version of the figure has been carefully incorporated into the manuscript. We believe these enhancements address the concerns raised and contribute to the overall clarity of the visual representation.  We appreciate your time and valuable insights. |
| Include some lines on the study's practical implications in the conclusion section. | Thank you for your valuable feedback. We have incorporated lines on the study's practical implications into the conclusion section as suggested. Please find the relevant additions in lines 474–481 and 482–486:  “In developed economies, investments in research and development are crucial for identifying methods to further utilise RES in climate mitigation. Concurrently, developing economies should prioritise increased investments in RE while minimising funds allocated to fossil fuels. Collaborations between governments of developing and developed economies are recommended to facilitate the sharing of financial resources and best practices for RE development [23, 42].”  “Furthermore, these findings also hold the potential to educate the general public on the pivotal role of RES in reducing CO2 emissions. Increased awareness in these areas is expected to drive positive changes in consumer behaviour and inspire innovative initiatives to address the effects of climate change.”  We believe these additions strengthen the practical implications of our study. Thank you for your insightful feedback. |
