# Supplementary material for: Renewable energy as a solution to climate change: Insights from a comprehensive study across nations
Source: PLoS One. 2024 Jun 20;19(6):e0299807. doi: 10.1371/journal.pone.0299807 (PMC11189203; doi:10.1371/journal.pone.0299807)
Supplement: Attachment — Submitted filename: Renewable Energy as a Solution to Climate.docx [file pone.0299807.s010.docx]

Thank you for allowing me to review your work. I believe this manuscript has potential to be published in PLOS ONE journal, but it requires significant improvements before submission. While the manuscript exhibits promise, it is crucial to address specific points to enhance the conceptual framework and logical coherence. Effectively addressing these areas will undoubtedly strengthen the overall quality of the document. ( "Renewable Energy as a Solution to Climate Change: Insights from a Comprehensive Study Across Nations" approaches.)

Comments

1.After reviewing this abstract, consider enhancing this abstract by incorporating sections on methods, results, and a dedicated conclusion for improved effectiveness.

2.Although the introduction is well-crafted, consider refining it by using the abbreviation (RE) for 'Renewable Energy.' Additionally, emphasize the concept of renewable energy projects and their potential to significantly reduce carbon emissions.

3.To improve the paper, this includes understanding what has been done, as well as what has not been done. Additionally, research questions and objectives should align with the purpose of the study.

4.While the authors adeptly address policy implications, it is recommended that they dedicate sufficient attention to articulating managerial implications. This addition would significantly contribute to enhancing the overall impact and effectiveness of their work. Additionally, you can take a look how this article has written managerial implication and limitation [**https://doi.org/10.3390/su151411289**](https://doi.org/10.3390/su151411289)
