# Supplementary material for: Renewable energy as a solution to climate change: Insights from a comprehensive study across nations
Source: PLoS One. 2024 Jun 20;19(6):e0299807. doi: 10.1371/journal.pone.0299807 (PMC11189203; doi:10.1371/journal.pone.0299807)
Supplement: Attachment — Submitted filename: Response to Reviewers.docx [file pone.0299807.s011.docx]

**Author Response Form**

Dear Editor and Reviewers,

We greatly appreciate the time invested in reading our manuscript and providing the necessary feedback to improve the overall quality of our work. All the mentioned comments have been considered and the revisions made accordingly.

Please note that the line numbers referred to in this document are aligned with the revised manuscript which includes track changes.

Thank you once again for your valuable feedback.

| **Comments** | **Author Response** |
| --- | --- |
| **Journal Requirements** | |
| Please review your reference list to ensure that it is complete and correct. If you have cited papers that have been retracted, please include the rationale for doing so in the manuscript text, or remove these references and replace them with relevant current references. Any changes to the reference list should be mentioned in the rebuttal letter that accompanies your revised manuscript. If you need to cite a retracted article, indicate the article’s retracted status in the References list and also include a citation and full reference for the retraction notice. | Well received with many thanks. The reference list was reviewed to ensure that no papers that have been retracted have been cited. |
| **Reviewer Comments** | |
| 1.If the authors have adequately addressed your comments raised in a previous round of review and you feel that this manuscript is now acceptable for publication, you may indicate that here to bypass the “Comments to the Author” section, enter your conflict of interest statement in the “Confidential to Editor” section, and submit your "Accept" recommendation.  Reviewer #2: All comments have been addressed  Reviewer #3: All comments have been addressed | Thank you for the comments that were provided helping us improve the overall quality of our manuscript. |
| 2. Is the manuscript technically sound, and do the data support the conclusions?  The manuscript must describe a technically sound piece of scientific research with data that supports the conclusions. Experiments must have been conducted rigorously, with appropriate controls, replication, and sample sizes. The conclusions must be drawn appropriately based on the data presented.  Reviewer #2: Yes  Reviewer #3: Yes | Well received with many thanks. |
| 3. Has the statistical analysis been performed appropriately and rigorously?  Reviewer #2: Yes  Reviewer #3: Yes | Duly noted with thanks. |
| 4. Have the authors made all data underlying the findings in their manuscript fully available?  The PLOS Data policy requires authors to make all data underlying the findings described in their manuscript fully available without restriction, with rare exception (please refer to the Data Availability Statement in the manuscript PDF file). The data should be provided as part of the manuscript or its supporting information, or deposited to a public repository. For example, in addition to summary statistics, the data points behind means, medians and variance measures should be available. If there are restrictions on publicly sharing data—e.g. participant privacy or use of data from a third party—those must be specified.  Reviewer #2: Yes  Reviewer #3: Yes | Thank you for your feedback. All the data sources that were used are openly available for use and any supporting information has been included in as supplementary files. |
| 5. Is the manuscript presented in an intelligible fashion and written in standard English?  PLOS ONE does not copyedit accepted manuscripts, so the language in submitted articles must be clear, correct, and unambiguous. Any typographical or grammatical errors should be corrected at revision, so please note any specific errors here.  Reviewer #2: Yes  Reviewer #3: Yes | Well noted with thanks. |
| **Reviewer #2** | |
| Thank you for allowing me to review your work. I believe this manuscript has potential to be published in PLOS ONE journal, but it requires significant improvements before submission. While the manuscript exhibits promise, it is crucial to address specific points to enhance the conceptual framework and logical coherence. Effectively addressing these areas will undoubtedly strengthen the overall quality of the document. ( "Renewable Energy as a Solution to Climate Change: Insights from a Comprehensive Study Across Nations" approaches.)  Comments | Thank you for taking the time to review our work and provide your feedback on how our manuscript could be improved. We have carefully read and incorporated all the necessary changes to our paper improving the overall quality of the document. |
| After reviewing this abstract, consider enhancing this abstract by incorporating sections on methods, results, and a dedicated conclusion for improved effectiveness. | Well noted. According to the author guidelines of PLOS ONE, the abstract has not been divided into sub-sections. Instead, the abstract is written as a whole and does include the methods, results and conclusion in lines 28 -30 as follows:  “We examined the extent of the impact of renewable energy on carbon dioxide over time using panel, linear, and non-linear regression approaches. The results of our analysis, revealed that the majority of countries with the exception of Canada, exhibited a downward trend, underscoring the potential of increasing renewable energy consumption as an effective method to reduce carbon dioxide emissions and combat climate change. Furthermore, to reduce emissions and combat climate change, it is advisable for nations with the highest carbon dioxide emissions to adopt and successfully transition to renewable energy sources.” |
| Although the introduction is well-crafted, consider refining it by using the abbreviation (RE) for 'Renewable Energy.' Additionally, emphasize the concept of renewable energy projects and their potential to significantly reduce carbon emissions. | Thank you for your feedback. The term Renewable Energy has been abbreviated using RE. The use of renewable energy projects to reduce carbon emissions has been added in the lines 89 - 94 as follows:  “Governments and other relevant stakeholders initiate RE related projects to smoothen the transition from non-RE to RE in certain industries. It has been proven that stakeholder engagement and their relationship with project management teams contributes significantly to the success of the project in countries such as Pakistan [29]. A high success rate for such projects will directly impact the CO2 emission levels around those industries.” |
| To improve the paper, this includes understanding what has been done, as well as what has not been done. Additionally, research questions and objectives should align with the purpose of the study. | Duly noted. While the literature review presents a detailed overview on past studies, we have also included the following sentences in lines 112 – 119 in the introduction to clarify the research gap:  “While previous research has focused on individual countries or a limited number of countries, there need to be more comprehensive global studies to provide a thorough understanding of the worldwide relationship between CO_2_ emissions and RE for a significant period. Therefore, this study provides a broader analysis based on a more substantial number of countries considering a more comprehensive period using the latest available data to obtain a more accurate depiction of the global status of the relationship between two variables helping for more precise decision-making.”  The research question and objective of the study have been mentioned in lines 115 – 119 as:  “Therefore, this study, satisfying the research question to understand what the impact of RE on CO_2_ emissions is, provides a broader analysis based on a more substantial number of countries considering a more comprehensive period using the latest available data to obtain a more accurate depiction of the global status of the relationship between two variables helping for more precise decision-making.”  “The study’s primary objective is to investigate the impact of RE on CO_2_ emissions in various countries …” |
| While the authors adeptly address policy implications, it is recommended that they dedicate sufficient attention to articulating managerial implications. This addition would significantly contribute to enhancing the overall impact and effectiveness of their work. Additionally, you can take a look how this article has written managerial implication and limitation https://doi.org/10.3390/su151411289 | Highly appreciate the feedback. Taking your comment and the structure of the paper suggested into consideration we have added an additional section on managerial implications in the lines 557-568 as mentioned below.  “The top 12 CO2 emitting countries can gain many insightful recommendations from this study. Apart from Canada, where there’s a positive relationship from REC to CO2, all 11 other countries can highly promote RE related products in their most revenue generating industries. Countries such as India and China can focus on implementing RE projects to their manufacturing and production industry. Initiatives such as these projects will lead to sustainable revenue generating industries while generating opportunities for the community in the form of project managers, project management teams etc. Project managers will be further inspired to develop strategic plans to initiate high value collaborations [29]. With the involvement of the government, investors and other external stakeholders, renewable energy projects can bring an immense amount of value to these countries in their journey to achieving sustainable development.” |
| **Reviewer #3** | |
| In the latest edition, the authors effectively addressed all necessary comments, resulting in a further enhancement of the paper's quality. | Thank you for your feedback and for taking the time to review our paper and provide precise recommendations to improve the overall quality of our work. |
